# Differential tropisms of old and new world hantaviruses influence virulence and developing host-directed antiviral candidates

**DOI:** 10.1371/journal.ppat.1013401

**Published:** 2025-08-26

**Authors:** Arjit Vijey Jeyachandran, Joseph Ignatius Irudayam, Swati Dubey, Nikhil Chakravarty, Maria Daskou, Anne Zaiss, Gustavo Garcia, Bindu Konda, Aayushi Shah, Aditi Venkatraman, Baolong Su, Cheng Wang, Qi Cui, Kevin J. Williams, Sonal Srikanth, Ashok Kumar, Yanhong Shi, Arjun Deb, Robert Damoiseaux, Barry R. Stripp, Arunachalam Ramaiah, Vaithilingaraja Arumugaswami

**Affiliations:** 1 Department of Molecular and Medical Pharmacology, University of California, Los Angeles, California, United States of America; 2 Department of Epidemiology, University of California, Los Angeles, California, United States of America; 3 School of Medicine, California University of Science and Medicine, Colton, California, United States of America; 4 Department of Medicine, Lung and Board of Governors Regenerative Medicine Institute, Cedars-Sinai Medical Center, Los Angeles, California, United States of America; 5 Department of Biological Chemistry, David Geffen School of Medicine, University of California, Los Angeles, California, United States of America; 6 UCLA Lipidomics Lab, University of California, Los Angeles, California, United States of America; 7 Department of Neurodegenerative Diseases, Beckman Research Institute of City of Hope, Duarte, California, United States of America; 8 Department of Physiology, David Geffen School of Medicine, University of California, Los Angeles, California, United States of America; 9 Department of Ophthalmology, Visual and Anatomical Sciences, Wayne State University, Detroit, Michigan, United States of America; 10 Division of Cardiology, Department of Medicine, David Geffen School of Medicine, UCLA, Los Angeles, California, United States of America; 11 Eli & Edythe Broad Center of Regenerative Medicine and Stem Cell Research, UCLA, Los Angeles, California, United States of America; 12 California NanoSystems Institute, UCLA, Los Angeles, California, United States of America; 13 Department of Bioengineering, Samueli School of Engineering, UCLA, Los Angeles, California, United States of America; 14 City of Milwaukee Health Department, Milwaukee, Wisconsin, United States of America; NIAID: National Institute of Allergy and Infectious Diseases, UNITED STATES OF AMERICA

## Abstract

Hantaviruses are zoonotically transmitted from rodents to humans through the respiratory route, with no currently approved antivirals or widely available vaccines. The recent discovery of interhuman-transmitted Andes virus (ANDV) necessitates the systematic identification of cell tropism, infective potential, and potent therapeutic agents. We utilized human primary lung endothelial cells, various pluripotent stem cell-derived heart and brain cell types, and established human lung organoid models to evaluate the tropisms of Old World Hantaan (HTNV) and New World ANDV and Sin Nombre (SNV) viruses. ANDV exhibited broad tropism for all cell types assessed. SNV readily infected pulmonary endothelial cells, while HTNV robustly amplified in endothelial cells, cardiomyocytes, and astrocytes. We also provide the first evidence of hantaviral infection in human 3D distal lung organoids, which effectively modeled these differential tropisms. ANDV infection transcriptionally promoted cell injury and inflammatory responses, and downregulated lipid metabolic pathways in lung epithelial cells. Evaluation of selected drug candidates and pharmacotranscriptomics revealed that the host-directed small molecule compound urolithin B inhibited ANDV infection and restored cellular metabolism with minimal changes in host transcription. Given the scarcity of academic BSL-4 facilities that enable *in vivo* hantaviral studies, this investigation presents advanced human cell-based model systems that closely recapitulate host cell tropism and responses to infection, thereby providing critical platforms to evaluate potential antiviral drug candidates.

## Author summary

Hantaviruses are fatal human pathogens that cause hemorrhagic fevers and are classified into either Old World or New World groups. Though most hantaviruses utilize zoonotic transmission, the New World Andes virus (ANDV) is unique in its ability to spread between humans. This distinct transmission mode underscores the need to investigate its cell tropism, pathogenicity, and therapeutic targets. Thus, we performed a systems-level comparison of the Old World Hantaan virus (HTNV) and New World hantaviruses, ANDV and Sin Nombre virus (SNV), using human lung, heart, and brain cell models, alongside lipidomic and transcriptomic profiling. We observed that ANDV exhibits broad tropism, infecting all tested cell types, including lung epithelial cells. HTNV replicated in lung endothelial, heart, and brain cells, whereas SNV replication was largely confined to lung endothelial cells. Notably, ANDV infection induced stronger host transcriptional changes, promoted cell injury and inflammatory responses, and suppressed lipid metabolic pathways in lung epithelial cells. Further drug testing and pharmacotranscriptomic analysis identified effective inhibitors of ANDV infection, including urolithin B, that restored cellular metabolism with minimal transcriptional disruption. This study provides a comparative framework for understanding hantavirus cell tropism and host responses and highlights potential antiviral candidates for treating these severe viral infections.

## Introduction

Hantaviruses have been major human pathogens of concern for several decades and are classified as Old World and New World hantaviruses. Hantaviruses first came to the world’s attention during the Korean War in the 1950s. Over 3,000 troops fell ill with Korean hemorrhagic fever, now referred to as hemorrhagic fever with renal syndrome (HFRS) [1] – the etiological agent of which is now known to be Old World Hantaan virus (HTNV), named after the Hantan River located near the outbreak sites. Hantaviruses (family *Hantaviridae*) are transmitted mainly by rodents, often mice, voles, and rats, and insectivores, such as shrews and moles, via aerosolized excrement – a distinguishing factor from the other members of the order *Elliovirales*, which are transmitted via arthropod vectors [2]. HTNV is transmitted by the rodent species *Apodemus agrarius*, which is primarily found in Central Europe, Russia, Korea, and China [3,4]. New World Sin Nombre virus (SNV) is carried by *Peromyscus maniculatus*, which inhabits the United States and Canada [5,6]. New World Andes virus (ANDV) is carried by *Oligozomys longicaudatus*, which resides in Argentina and Chile along the Andes mountains [3,7,8]. Most hantavirus species have not been detected to be transmitted between humans. Thus, their main mode of transmission is through the animal reservoirs. Several highly pathogenic viral species have been identified in recent decades, causing HFRS (e.g., Puumala virus [PUUV] [9] and HTNV) or hantavirus cardiopulmonary syndrome (HCPS) (e.g., SNV [5] and ANDV [10]). HCPS can also be referred to as hantavirus pulmonary syndrome (HPS) [11]. There are also low- and non-pathogenic hantavirus species, such as Tula virus and Prospect Hill virus [12]. Depending on the infecting strain, the mortality rate can vary from 1% to 40% [13]. One study tracking the ANDV outbreak in Epuyén, Argentina, reported that the median reproductive number when ANDV spread was left uncurbed was 2.12 new cases per infected person, but was reduced to 0.93 when infection control measures were implemented [14]. Since ANDV has shown evidence of human-to-human transmission, it is a viral disease of major concern [14–16]. With hantaviruses again being thrust into the worldview, having caused the recent death of Betsy Arakawa, the wife of the late renowned actor, Gene Hackman, in Santa Fe, New Mexico [17], as well as three additional deaths in California’s Mammoth Lakes [18], there is a clear and present need to better understand, characterize, and treat these devastating viruses. As such, the scope of this study primarily focuses on the Old World HTNV and the New World SNV and ANDV, as these viral species pose a significant threat to the global population.

Hantaviruses contain an 11–13 kb long, tri-segmented, negative-sense, single-stranded RNA genome split into a small (S), medium (M), and large (L) segment within an envelope covered in viral glycoproteins [19]. Hantavirus Gn/Gc glycoproteins are the only viral proteins exposed on the surface of virions, which facilitate virus attachment and entry to the cells. Although the overall architecture of the Gn/Gc complex is conserved among hantaviruses, Gn is less conserved than Gc and is proposed to bind to cell-surface receptors during viral entry. Due to their variability in Gn sequence, distinct hantavirus clades are likely to use different attachment factors and/or receptors [20–22]. While there is still much uncertainty as to the extent of cell types hantaviruses can infect, there is evidence that the danger of hantaviruses comes from their ability to infect a wide variety of cells across several different vital systems – namely, the renal, pulmonary, nervous, and cardiac systems [13]. Evidence using animal infection studies, post-mortem analyses of infected human tissue, and cell culture systems has been presented that some species of hantaviruses can readily infect endothelial [23] and mononuclear [24–31] cells through a less-understood mechanism and spread throughout the human body to nearly all major organs [23]. Other *in vitro* studies have shown that hantaviral species can infect several immune cell types, hepatocytes, Kupffer cells, kidney cells [32,33], pharynx, and submaxillary glands [23,32–35].

Despite extensive and intensive research efforts, no effective prophylactic or therapeutic host-directed or direct-acting antiviral agents have been approved targeting hantaviruses in the United States. Although Hantavax is the best-known approved prophylactic targeted HTNV, it is only approved for use in Asia. Virus-like particle, inactivated, recombinant, viral vector, and nucleic acid-based vaccine candidates have been developed and are either currently being tested or undergoing clinical trials with varying ability to prevent disease caused by HTNV, PUUV, SEOV, ANDV, and DOBV [36]. The drug vandetanib demonstrated semi-prophylactic efficacy in ANDV-infected Syrian hamsters, delaying lethality and increasing total survival by 23% when administered five days prior to the ANDV challenge [37]. Several antiviral drug candidates have also been investigated for their effects in treating hantaviral disease. Ribavirin, although promising in mouse and Syrian hamster models [38–41], was generally not found to be effective in humans during clinical trials [42–44]. Other drug candidates, such as lactoferrin, 1-beta-d-ribofuranosyl-3-ethynyl-[1,2,4]triazole (ETAR), and favipiravir, have been tested in animal models, showing some efficacy in treating hantavirus infection [45].

It has been extensively demonstrated that both Old and New World hantaviruses readily infect endothelial cells (ECs) [46–49]. *In vitro* studies have shown that various Old World viruses, including HTNV, PUUV, SEOV, Prospect Hill virus, and Leakey virus, can actively infect human umbilical vein endothelial cells [50]. Post-mortem analysis of HCPS patients infected with the New World SNV revealed a widespread presence of hantaviral antigens in the lungs, specifically in pulmonary parenchymal cells, hematological cells, and reticuloendothelial cells [27,51]. ANDV has also been shown to infect lymphatic and lung microvasculature endothelial cells **in vitro* [*52,53]. However, despite extensive studies, the cellular and molecular mechanisms underlying the pathophysiology of hantaviral disease are still poorly understood.

There is a lack of comparative data analyzing the replication and host responses between New and Old World hantaviruses. Thus, we aim to systematically assess differences in viral tropism between Old and New World hantaviruses in lung, heart, and brain cell types. In this study, we developed human pluripotent stem cell (hPSC)-derived cellular and organoid systems to model and investigate differences in pathogenic processes at the molecular level, as well as cell injury mechanisms. Utilizing the developed infectious cell culture system, we have evaluated antiviral agents targeting ANDV and their pharmacotranscriptomic profile.

## Results

### New world ANDV displays tropism for both pulmonary endothelial and lung epithelial cell types

**Fig 1:**
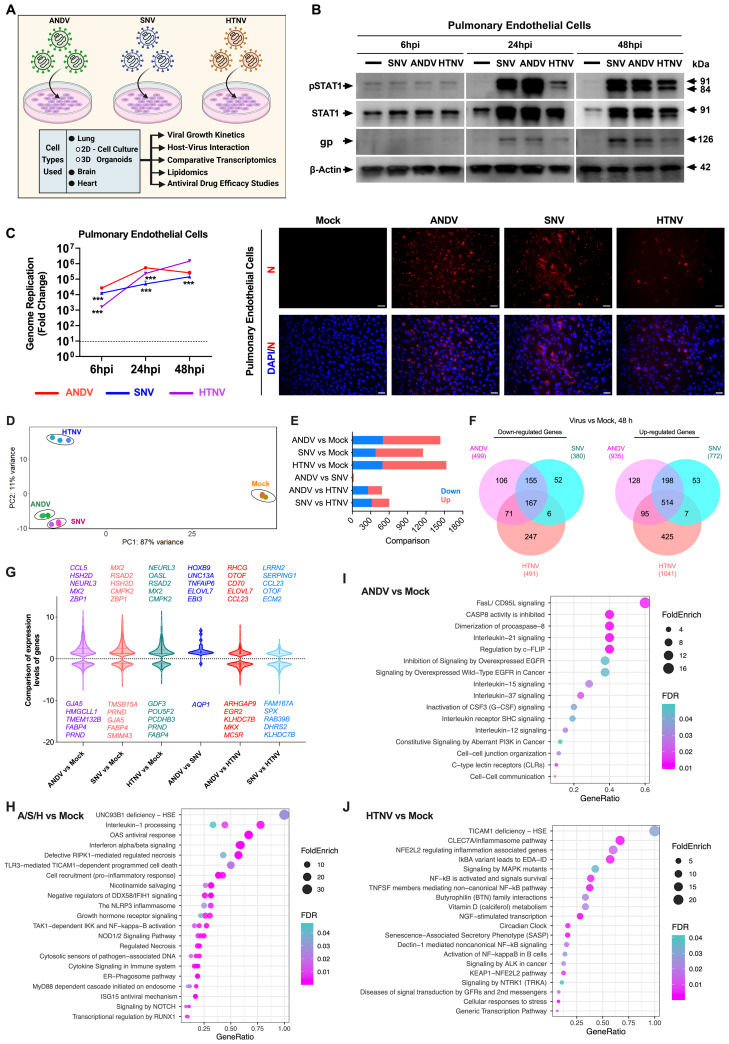
Pulmonary endothelial cell tropism of various hantaviruses. **A)** Schematic outline of cell types used, and methodologies employed in this study. Key assays were conducted for selected cell types. Image created on BioRender. **B)** Western blot analysis of pulmonary endothelial cells infected with hantaviruses at indicated timepoints. Viral envelope antigen was detected using Glycoprotein 1 (gp) Antibody. **C)** The graphs demonstrate the differing levels of viral genome replication of hantaviruses in pulmonary endothelial cells. Statistical analysis (student t-test) was performed as compared with ANDV-infected cells (left). Immunofluorescence assay (IFA) analysis of these infected cells detected viral nucleocapsid (red) at 48 hours post-infection (hpi) (right). Scale bar: 25μm. Student t-test was performed as compared with ANDV-infected cells: ***, P < 0.001. Representative data presented from two experimental repeats. **D)** Unsupervised PCA was performed using normalized RNA-seq data from human pulmonary endothelial cells infected with HTNV (blue), ANDV (green), SNV (magenta), and mock-treated (orange) for 48 hours. PC1 and PC2 account for 87% and 11% of the total variance, respectively. **E)** Bar plot shows the number of significantly downregulated (blue), and upregulated (red) genes in pulmonary endothelial cells infected with indicated viruses. **F)** Venn diagrams illustrate the number of common and distinct genes downregulated (left) and upregulated (right) in cells infected with ANDV (pink), SNV (blue), or HTNV (salmon), compared to mock. The central overlapping regions show genes commonly differentially expressed across all three hantavirus infections, while the outer sections highlight virus-specific DEGs. **G)** Violin plot shows patterns of differential gene expression levels (padj ≤ 0.01 with log2 Fold Change 1) in pulmonary endothelial cells across hantavirus infections. The 5 most down- or up-regulated DEGs are displayed. **(H-J)** Dot plots depict most overrepresented Reactome pathways among upregulated genes at 48 hpi in virus-infected pulmonary endothelial cells. H) Common pathways shared by all three viruses: ANDV (A), SNV (S) and HTNV (H). Pathways exclusively upregulated by ANDV and HTNV are illustrated in I) and J), respectively. The comprehensive list of pathways is provided in S2 Table. Pathways were identified through pathway enrichment analysis. Dot size reflects fold enrichment, while color indicates false discovery rate (FDR). GeneRatio represents the proportion of upregulated genes associated with each pathway.

Given that hantaviruses are well-known to spread via the respiratory route, we first aimed to systematically assess the susceptibility of various lung cell types to phylogenetically related Old and New World hantaviruses (S1A Fig, S1 Table). As such, we established human cell culture model systems, including a 3D distal lung organoid. We used Old World HTNV (Fojnica strain) as well as New World ANDV (Chile-9717869 strain) and SNV (SNV-77734 strain) for infection studies, as illustrated in Fig 1A. All three viruses were amplified in Vero E6 cells and replicated at similar levels (S1B Fig). Virus replication was quantified using strain-specific primer sets using sensitive RT-qPCR (S1B Fig; refer to Methods).

Since the New World hantaviruses cause cardiopulmonary diseases, we first examined the susceptibility of lung endothelial and epithelial cell types. Pulmonary endothelial and Calu-3 lung epithelial cells were infected with ANDV, SNV, and HTNV viruses at an MOI of 0.1. At 6, 24, and 48 hours post-infection (hpi), cellular RNA was collected, and viral load was assessed by RT-qPCR.

We observed ready replication of all three hantaviruses in pulmonary endothelial cells. Western blot analysis revealed that both New World HCPS-causing viruses (ANDV and SNV) had similar levels of viral glycoprotein (gp) expression and increased levels of total and phosphorylated forms of STAT1, indicating activation of the type I interferon (IFN) antiviral signaling pathway (Fig 1B). We observed a subdued level of phospho-STAT1 in HTNV-infected pulmonary endothelial cells, corresponding to lower levels of viral glycoprotein detection. RT-qPCR analysis showed that ANDV had the highest levels of genome replication at 6 hpi, peaking at 24 hpi (Fig 1B, 1C). In comparison, SNV exhibited a steady increase in infection over the study course, with genome replication levels at 6 hpi similar to those of ANDV, whereas HTNV showed viral replication levels peaking at 48 hpi (Fig 1C). Immunofluorescence analysis (IFA) showed viral nucleocapsid (N) protein detected in all virus-infected cells after 48 hpi (Fig 1C). It is important to note that, given that the N protein polyclonal mouse antibody used is specific for SNV nucleocapsid, this antibody may show varying sensitivity in detecting all three hantaviruses by immunofluorescence analysis. This consideration should be noted in further analysis of other cell types.

To further explore the molecular basis of cell injury caused by these pandemic potential viruses and associated host responses, virus-specific transcriptional changes in infected pulmonary endothelial cells were investigated. Bulk RNA sequencing analysis was performed on samples collected at 48 hpi. Unsupervised principal component analysis (PCA) of gene expression levels revealed that the infected samples clustered by each viral group, distinct from the uninfected mock samples (Fig 1D). Moreover, this analysis revealed that the most significant variations between the viral-infected samples were that the New World hantaviruses ANDV- and SNV-infected samples clustered adjacent to each other, while Old World hantavirus HTNV-infected samples were distinctly apart, indicating that host transcriptional responses to New World hantaviruses are similar (Fig 1D-1G). This was further supported by a greater number of commonly differentially expressed genes (DEGs) between ANDV and SNV that were downregulated (n = 322) and upregulated (n = 712) (Fig 1F), suggesting that several host molecular functions were commonly dysregulated by the two New World viruses. The total number of host genes dysregulated by ANDV, SNV, and HTNV was 1,434, 1,152, and 1,532, respectively. When evaluating virus-specific overlaps, we found that only 167 downregulated and 514 upregulated genes were commonly dysregulated by all three viruses. 52 (SNV), 106 (ANDV), and 247 (HTNV) of these were uniquely downregulated DEGs, while 53 (SNV), 128 (ANDV), and 425 (HTNV) were distinctively upregulated (Fig 1F). Further comparison among New World hantaviruses also revealed that host responses to ANDV and SNV were similar (25 DEGs) (Fig 1E). In contrast, comparison of New World viruses ANDV and SNV with Old World hantavirus (HTNV) increased the host responses approximately 19- and 24-fold, respectively.

The most significantly upregulated and downregulated genes were further annotated for each virus based on their expression level (Fig 1G). A total of 514 genes, including *MX2, NEURL3, RSAD2, CCL5*, *ZBP1, OASL, IFI16, PARP9,* and *DTX3L,* were found to be commonly upregulated in endothelial cells across all three hantaviral infections. Most of these genes were found to be involved in inflammatory and innate immune signaling responses. Genes *RTP4, ATP10A, EPSTI1, BATF2, TYMP,* and *SLC15A3* were also found to be commonly upregulated. Genes *FABP4, FABP5,* and *FABP5P7*, which encode cytoplasmic fatty acid-binding proteins facilitating fatty acid uptake, transport, and metabolism, were significantly downregulated. To understand the potential biological implications of hantavirus-induced transcriptional changes, we performed Reactome pathway analysis using these DEGs. The common pathways upregulated upon infection by each of these three viruses included innate antiviral and inflammatory pathways, such as OAS and ISG15-mediated antiviral responses, interferon α/β signaling, cytokine and interleukin signaling, NOD1/2 signaling, and the NLRP3 inflammasome pathway (Fig 1H; S2 Table). Additionally, programmed cell death and regulated necrosis pathways were also upregulated in infected cells. Upon conducting a further detailed analysis, we found that ANDV and HTNV had several uniquely enriched pathways (Fig 1I and 1J, and S2 Table), whereas SNV induced very few unique pathways. ANDV exclusively induced the FasL/CD95L, as well as multiple interleukin signaling pathways, whereas HTNV induced pathways such as the CLEC7A/inflammasome pathway, circadian clock, and senescence-associated secretory phenotype (Fig 1I and 1J). Regarding downregulated pathways, HTNV only downregulated the extracellular matrix organization pathway, whereas SNV and ANDV downregulated pathways involved in virus-induced host shutoff, including ribosomal scanning and start codon recognition, cap-dependent translation initiation, and eukaryotic translation elongation, as well as metabolism of amino acids and derivatives.

**Fig 2:**
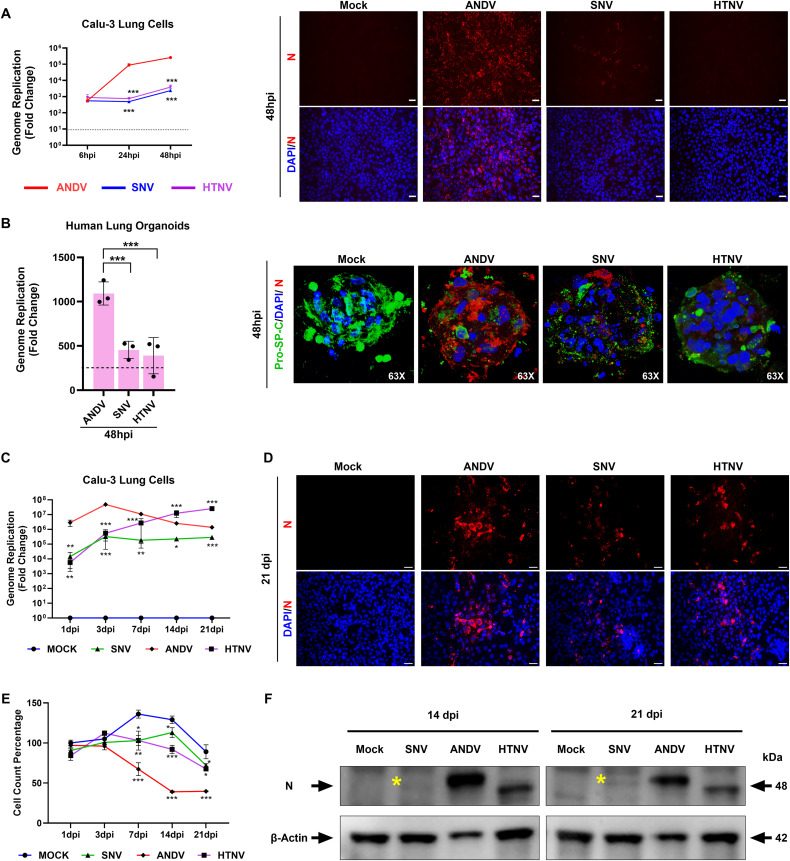
Infection studies in Calu-3 lung epithelial cells and human distal lung 3D organoids. **A)** The graphs demonstrate the differing levels of viral genome replication of hantaviruses in pulmonary endothelial and Calu-3 cells at 6-, 24-, and 48-hours post-infection (hpi), measured by RT-qPCR. Statistical analysis (student t-test) was performed as compared with ANDV-infected cells (left). Immunofluorescence assay (IFA) analysis of these infected cells detected viral nucleocapsid (N, red) and nuclei (DAPI, blue) at 48 hpi (right). Scale bar: 25μm. Representative data presented from three experimental repeats. **B)** The graph shows the levels of viral genome replication in human distal lung 3D organoids at 48 hpi (left). Confocal microscopy images of human distal lung 3D organoids depict lung marker pro-SP-C (green) and hantaviral nucleocapsid (red) (right). Scale bar: 50μm. **C)** The graph shows the fold change in hantaviral genome replication from 1, 3-, 7-, 14- and 21-days post-infection (dpi) in Calu-3 cells, as measured by RT-qPCR. Statistical analysis (Student’s t-test) was performed in comparison to ANDV-infected cells. **D)** IFA analysis of infected cells detecting viral nucleocapsid (red) at 21 dpi. Scale bar: 20µm. Both SNV and HTNV established robust infection by Day 21. **E)** Graph shows cell count percentage at various time points for both hantavirus-infected and uninfected cells. Student t-test was performed as compared to mock. **F)** Western blot analysis of Calu-3 cells infected with hantaviruses at indicated time points. Viral Nucleocapsid protein (N) was detected using nucleocapsid antibody. Asterisks indicate the detected N protein band for SNV. Statistical analysis was performed using Student t-test (*, P < 0.05; **, P < 0.01; ***, P < 0.001). Representative data presented from two experimental repeats.

We then wanted to investigate the potential of these viruses to infect lung parenchymal, or epithelial, cells. In Calu-3 lung epithelial cells, we observed that ANDV established a robust infection with a genome replication rate over 100-fold higher than that of SNV or HTNV (Fig 2A). This observation was further supported by IFA detecting viral N protein (Fig 2A). Since Calu-3 is a lung adenocarcinoma cell line, we further investigated the hantaviral tropism of lung epithelium using a primary human distal 3D lung organoid model (Fig 2B). Through this model system [54], we observed that ANDV replicated at a higher level compared to HTNV and SNV, which was similar to the responses we noted in Calu-3 cells (Fig 2A). The presence of alveolar type 2 (AT2) cells within distal lung organoids was confirmed by staining for the AT2 cell marker, Pro-SP-C (Fig 2B). In general, the SNV strain used exhibited poor replication efficiency in all tested cell systems based on qPCR analysis and further confirmed by immunostaining, except in pulmonary endothelial cells.

Since the levels of SNV and HTNV replication are lower at the 48-hour timepoint in Calu-3 cells compared to ANDV, we postulate that SNV and HTNV have slower growth kinetics in lung epithelial cells and may establish active infections after multiple rounds of proliferation. Thus, we infected the cells with these various viruses at a low MOI of 0.01 to allow the virus to replicate for multiple weeks. The cells had half media change every other day. Cell lysates were harvested on Days 1, 3, 7, 14, and 21 post-infection. RT-qPCR analysis showed that ANDV exhibited the highest levels of genome replication at 3 dpi and persisted up to the Day 21 endpoint (Fig 2C). In contrast, HTNV showed a gradual increase in genome replication up to Day 21 (Fig 2C). However, SNV replication peaked at Day 3 and remained steady until Day 21 (Fig 2C). SNV- and HTNV-positive cells were observed at later timepoints via IFA analysis (Fig 2D). Notably, we also observed ANDV-positive cells at all tested timepoints. To understand if hantaviral infections have any adverse effects on cell growth and survival, we quantified cell density at each time point (Fig 2E). All viruses were observed to have a significant impact on cell growth dynamics as compared to mock-infected cells. ANDV infection resulted in the loss of over 50% of the cells during the course of this study. Due to low-grade persistent infection throughout all time points, SNV was also seen to induce a significant decrease in cell growth over time. Similarly, HTNV caused cell growth arrest by 7 dpi. Upon analyzing Western blots, we found that viral-specific nucleocapsid protein was expressed at varying levels among the viruses (Fig 2F). For both SNV and HTNV, viral nucleocapsid antigens were detected after 14 dpi. These observations suggest that HTNV and SNV can establish chronic-persistent infections in lung epithelial cells *in vitro*.

### HTNV and ANDV display strong heart and brain cell tropisms

**Fig 3:**
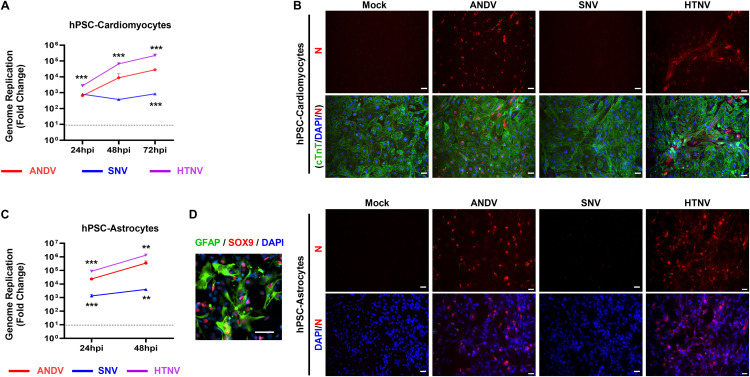
Cardiac and brain cell tropism of various hantaviruses. **A)** Graph indicates the fold change of hantaviral genome replication in human iPSC-cardiomyocytes. **B)** Immunofluorescent analysis detects viral nucleocapsid (red) and cardiac troponin (green) in hantavirus-infected iPSC-cardiomyocytes at 48 hours post-infection (hpi). Scale bar: 25μm. **C)** Graph shows genome replication in human iPSC-astrocytes upon infection with various hantaviruses. **D)** Immunofluorescent analysis detects (left panel): GFAP (green) and SOX9 (red) and (right panel): viral nucleocapsid (red) in hantavirus-infected iPSC-astrocytes at 48 hpi. Scale bar: 25μm. Representative data presented from two experimental repeats. Student t-test was performed as compared with ANDV-infected cells: *, P < 0.05; **, P < 0.01; ***, P < 0.001.

After evaluating the pulmonary tropisms of these viruses, we focused our efforts on gaining a better understanding of hantaviral infection in heart cells using human pluripotent stem cell-derived cardiomyocytes (hPSC-CMs). qPCR analysis showed that both ANDV and HTNV replicated at a higher level, whereas SNV showed no amplification from baseline (Fig 3A). This finding was further confirmed by IFA, with SNV showing levels of N protein below detectable limits (Fig 3B). Subsequently, to assess host innate immune and inflammatory responses, we evaluated the gene expression levels of OAS1, IFN-λ (lambda), IFN-β, and IL1-β in both hPSC-CMs and Calu-3 cells (S1D Fig). Our observations revealed a significant upregulation of these genes over time in response to ANDV infection in both cell types (S1D Fig). At the functional level, ANDV-infected hPSC-cardiomyocytes had significantly reduced cell-beat contractions – the motion of the cells as they contract and lengthen their cell bodies to simulate the pumping of blood through the heart (S1C Fig).

Though rare, hantaviruses have also been shown to cause neurological symptoms and disease [55–58]. As such, we further investigated the infectivity of these viruses in human astrocytes, a major brain cell type (Fig 3C, 3D). Mouse model studies using HTNV showed brain infection and resulting neurological signs, such as paralysis [59,60]. We observed that both ANDV and HTNV established active infection in hPSC-derived astrocytes (hPSC-astrocytes) (Fig 3C, 3D). This was further confirmed by IFA analysis recognizing viral N protein (Fig 3D). Altogether, we observed that ANDV-infected cells demonstrated increased levels of immune gene activation, as well as increased virulent activity in these cell types.

### Comparative transcriptomics analysis of old and new world hantaviruses in lung, heart and brain cell types

**Fig 4:**
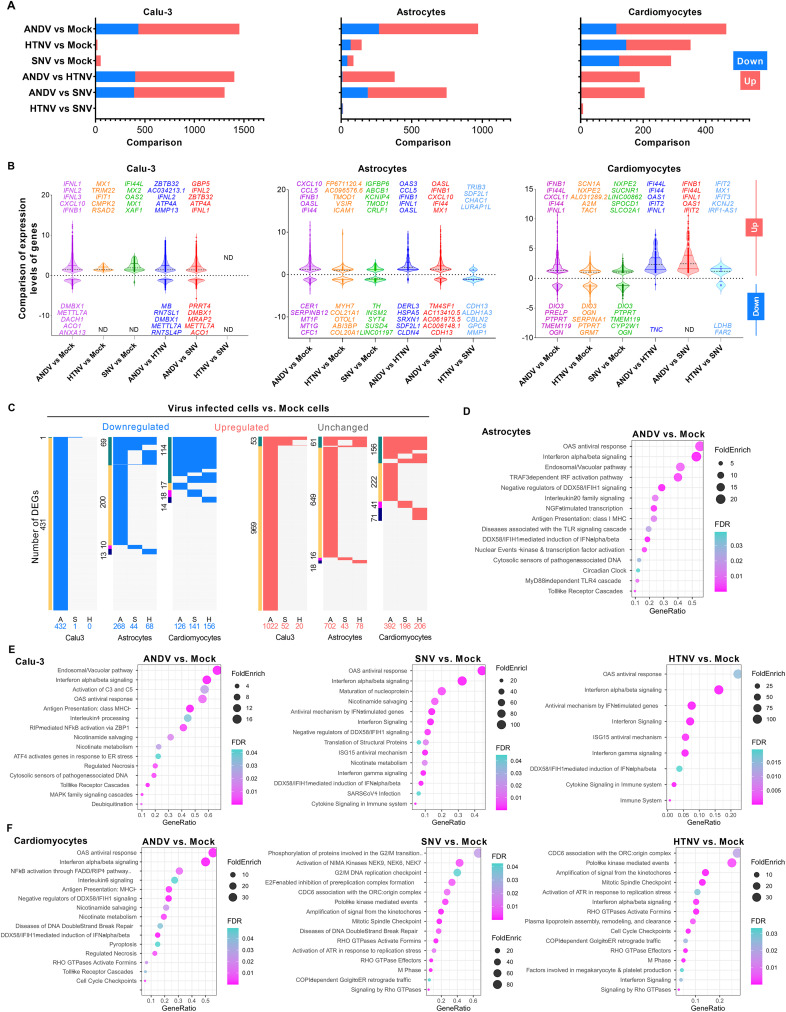
Comparative transcriptomic analysis of hantaviruses. **A)** Bar graph shows the total number of down- and up-regulated genes in Calu-3, hPSC-astrocyte and hPSC-CM cells infected with indicated viruses. **B)** Violin plots show a comparison of expression levels (log2 Fold Change) of differentially expressed genes (DEGs) in various samples. **C)** Comparison of down- and up-regulated DEGs in cells infected with indicated viruses (A, ANDV; S, SNV; H, HTNV). The sidebars show the DEGs group (green: DEGs shared by at least two datasets; others: exclusive to each dataset). **D)** The dot plot shows the up-regulated pathways in ANDV-infected hPSC-astrocytes. **E, F)** Dot plots represent the up-regulated pathways in infected Calu-3 and hPSC-CMs, respectively.

Using systems-level transcriptomics, we investigated host cellular responses and associated dysregulated pathways in response to Old and New World hantaviral infections of lung, heart, and brain cell types (Fig 4A). We used Calu-3 cells, hPSC-CMs, and hPSC-astrocytes infected with each virus (MOI 0.1). RT-qPCR analysis confirmed ANDV and HTNV genome replication in these cell types (Figs 2A and 3). However, SNV failed to establish active replication in hPSC-CMs and hPSC-astrocytes. For RNA sequencing analysis, samples collected at 48 hpi were used. Transcriptomics analysis of virus-infected and uninfected cell types revealed that ANDV-infected cell types exhibited a greater number of differentially expressed genes (DEGs) compared to HTNV and SNV (Fig 4A). HTNV- and SNV-infected cells show similar transcriptional responses, though significantly different from ANDV-infected cells. For instance, the expression of host genes in ANDV-infected lung cells was increased about 73- and 27-fold compared to HTNV- and SNV-infected cells, respectively, indicating viral species-specific differences in molecular dysregulation and associated host cell responses (Fig 4A-4C). Given the different infection rates of these viruses in lung cells, we anticipated that HTNV and SNV, with a lower percentage of infection, would not elicit as many DEGs as ANDV. However, because this is a bulk RNA-Seq, the transcriptional responses of both infected and uninfected bystander cells will be averaged out. This limitation may apply to subsequent analyses with different cell types and varying infection rates. The ANDV-infected hPSC-astrocytes also exhibited increased transcriptional responses (7-fold to HTNV; 11-fold to SNV). However, no significant transcriptional differences were observed when comparing these three viruses following infection of hPSC-CMs, reflecting cell-specific differences in tropism. These data also illustrate that, while lung cells, followed by brain cells, are more susceptible to ANDV, heart cells likely support the replication of ANDV and HTNV viral species, albeit at different levels. HTNV- and SNV-infected cells exhibited similar gene expression patterns, indicating that infection with either of these two viruses had a minimal effect, regardless of cell type, at 48 hpi. Comparison of ANDV- with HTNV- or SNV-infected cells displayed a higher number of upregulated genes [Calu-3: 1003 (ANDV vs. HTNV) and 915 (ANDV vs. SNV); astrocytes 370 (ANDV vs. HTNV) and 558 (ANDV vs. SNV); hPSC-CMs 188 (ANDV vs. HTNV) and 205 (ANDV vs. SNV)], suggesting that the pathogenic mechanism of virulent ANDV may differ at the genetic level relative to the other two viruses (Fig 4B, 4C). Comparison of HTNV-infected with SNV-infected cells revealed no noticeable difference in transcriptional responses in all host cell types. It is important to consider that these findings may also be attributed to lower replication levels of SNV and HTNV compared to ANDV, as these two strains share similarities in their zoonotic transmission, lacking the ability to transmit between humans (S1A Fig). Overall, the host transcriptional responses to each of the three hantaviruses were differential in nature, showing a range of only 19–79 commonly downregulated and 18–78 upregulated genes, suggesting that genes involved in a few biological functions are commonly dysregulated by these viruses in the tested cell types. Reflecting the *in vitro* experimental data, transcriptome data from three cell types also revealed that ANDV infection had the largest number of uniquely downregulated (Calu-3: 431; astrocytes: 200; hPSC-CM: 17) and upregulated (Calu-3: 969; astrocytes: 649; hPSC-CM: 222) genes (Fig 4C) compared to uninfected cells. Our results suggest that these genes may be viral species-specific responses induced by different cell types.

We identified 316 commonly upregulated genes (*FDR* 0.01) across three human cell types infected with ANDV (Fig 4C, S3 Table). Of these 316 genes, 20 were highly upregulated (>5 Log2FoldChange) in these cell types, most of which were related to innate immune and inflammatory chemokines (S3 Table). Similarly, these human cell types infected with HTNV had six commonly upregulated genes (*TAP1, DTX3L, DUSP1, PARP9, IFI16, REC8*), whereas those infected with SNV had three (*PLSCR1, DTX3L, IFI16*). Genes *DTX3L* and *IFI16* were found to be commonly induced across all three hantaviral infections. *PLSCR1* transcriptional induction was shared by both New World hantaviruses. These upregulated biomarkers may reflect the differential susceptibility and host responses of the tested cell types to these viruses.

The cell tropism of these viruses can also be determined by the level and type of viral entry receptors expressed by these cell types. Among previously described hantaviral entry receptor genes [3,14–16,20–22,61–72], six were transcriptionally stimulated by hantaviral infection in the three cell types studied (S4 Table). We identified ICAM1 to be commonly upregulated in all three cell types upon ANDV infection, whereas it was only upregulated in two cell types (hPSC-CMs and -astrocytes) upon HTNV infection and only in hPSC-astrocytes upon SNV infection. ICAM1 was also found to be upregulated during inflammatory response. GRIK1 was downregulated in hPSC-CMs and -astrocytes infected with hantaviruses. ANDV infection showed upregulation of cell type-specific viral entry genes (Calu-3: *CD55* and *ITGB3*; hPSC-astrocytes: *MERTK* and *VEGFA*). HTNV infection upregulated the *HAVCR2* gene in hPSC-astrocytes. However, the major cell receptor PCDH1 was not observed to be differentially regulated upon infection. Further exploiting the identified viral entry receptors and their interactions with viruses would be an attractive target for new antiviral therapeutics and vaccine technologies.

Pathway and Gene Ontology enrichment analyses of infected Calu-3 cells revealed the upregulation of immune pathways, including the OAS antiviral response, IFN-α/β signaling, IFN-γ signaling, and the ISG15 antiviral mechanism, by all three viruses. However, cell death mechanisms, including programmed cell death, death receptor signaling, and necrosis (Fig 4E), immune pathways such as many interleukin signaling and TLR cascades, and the MyD88-independent cascade were upregulated only by ANDV. These molecular signatures suggest that, compared to SNV and HTNV viruses, ANDV infection, which is generally more pronounced, also induces more pronounced inflammatory changes in lung epithelial cells in cell culture conditions. In hPSC-astrocytes, no pathways were upregulated by SNV and HTNV infection, while many immune pathways were upregulated by ANDV (Fig 4D). Pathway analysis of hPSC-CMs showed that upregulation of cell cycle pathway genes was common across all three viruses. In contrast, cell repair and related pathways were only upregulated in SNV and HTNV (Fig 4F). In contrast, ANDV infection upregulated many immune and inflammatory pathways, as well as cell death and necrosis pathways, in hPSC-CMs (S2A-S2D Fig). Taken together, our comprehensive transcriptome analysis revealed that i) all three viruses were able to modify the cell cycle at the transcriptional level, ii) enriched pathophysiological molecular changes occurred in all three cell types infected with ANDV compared to the other two viruses, and iii) ANDV infection may lead to lung and heart cell injury and cellular death.

### Cholesterol pathway is downregulated upon ANDV infection

**Fig 5:**
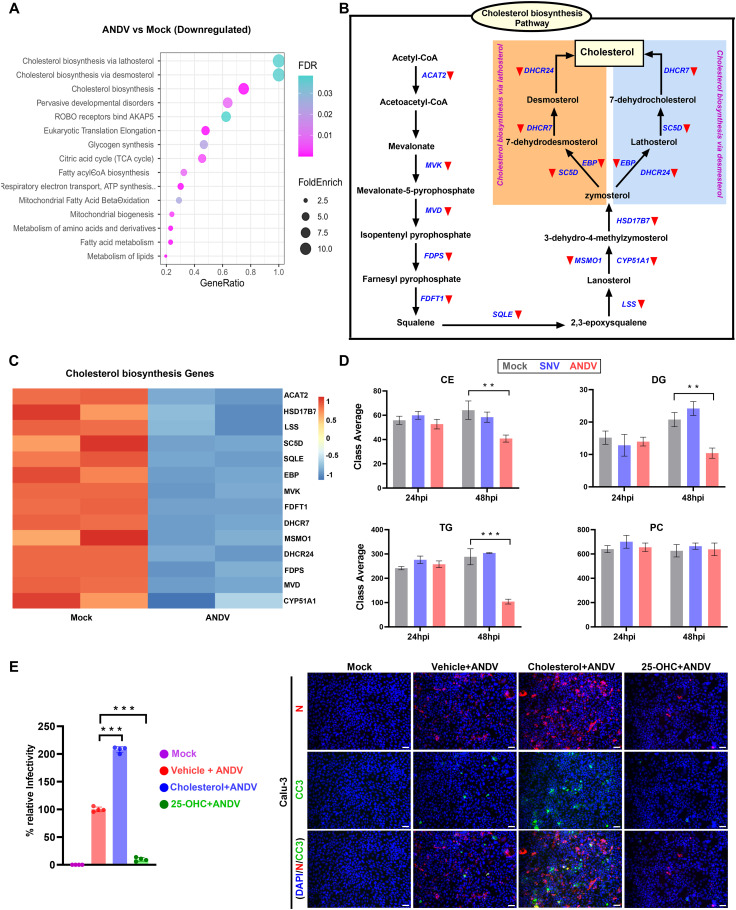
Cholesterol pathway dysregulation in ANDV-infected Calu-3 cells. **A)** The dot plot illustrates downregulated pathways, including cholesterol synthesis in ANDV-infected cells relative to uninfected cells at 48 hpi. **B)** The diagram illustrates the downregulated genes in the cholesterol synthetic pathway. **C)** Heatmap shows Z scores as expression levels of the genes involved in the cholesterol biosynthesis pathway. Blue and red colors represent downregulated and upregulated genes, respectively. **D)** Graphs indicate levels of lipid molecule subclasses at 24 hpi and 48 hpi. **E)** Graph shows the percent infectivity of ANDV at 48 hpi in indicated treatment conditions. Immunofluorescent analysis of ANDV-infected cells supplemented with cholesterol (100µM) or 25-OHC (10µM) at 48 hpi. Nucleocapsid = red; Cleaved caspase-3 = green. Scale bar: 25μm. Quantitative data are presented as mean ± standard deviation. Statistical analysis was performed using ANOVA, followed by Tukey’s post hoc test (*, P < 0.05; **, P < 0.01; ***, P < 0.001).

Upon identifying these transcriptional pathways, we sought to determine the metabolic pathways dysregulated during ANDV infection. Our transcriptomic analysis indicated that ANDV infection significantly downregulated the cholesterol synthesis pathways in Calu-3 cells and hPSC-astrocytes (Figs 5A, S3A). Genes controlling the key steps in cholesterol biosynthesis, such as *FDPS, ACAT2, MVD, LSS,* and *FDFT1*, were transcriptionally commonly suppressed upon viral infection at 48 hpi (Figs 5B-5C, S3B). This finding is surprising because ANDV has been reported to require cholesterol for viral entry and replication [68]. In the context of host-virus interactions, this observation may be due to the host’s antiviral response, which depletes cholesterol in infected cells to restrict virus production. However, this depletion can have the potential to damage cellular processes and thus play a role in hantaviral pathogenesis.

We conducted a lipidomics study to verify these transcriptional changes in cholesterol and lipid metabolites within infected cells. We observed that ANDV-infected Calu-3 cells had significantly lower levels of cholesterol esters, triglycerides, and diglycerides at 48 hpi (Fig 5D). SNV infection of Calu-3 cells did not affect the levels of these metabolites. Phosphatidylcholine (PC) and phosphatidylethanolamine (PE) levels were not affected in both ANDV- and SNV-infected cells. However, levels of their intermediate metabolites, lysophosphatidylcholine (LPC) and lysophosphatidylethanolamine (LPE), were increased upon ANDV infection (S3C Fig). We also observed reductions in triglyceride (TG) bonds and TG carbons in ANDV-infected cells (S3D Fig). Therefore, although possibly attributable to differences at baseline infection, this reduction in TG bonds and TG carbons likely confirms the viral impact on host cell lipid metabolism by dysregulating the processes involved in triglyceride synthesis.

Since cholesterol is important for ANDV replication, we performed a study in which cholesterol was supplemented in the cell media of ANDV-infected Calu-3 cells. We observed that supplementary cholesterol significantly enhanced ANDV infection (Fig 5E). This experiment confirmed that the depletion of cholesterol pathway-associated genes and metabolites observed at both the transcriptional and metabolomic levels is likely induced by the host’s antiviral response to ANDV infection. Furthermore, we observed that the gene *CH25H*, which encodes for the IFN-stimulated enzyme cholesterol 25-hydroxylase, was upregulated in ANDV-infected cells. This gene produced the antiviral metabolite 25-hydroxycholesterol (25-OHC). As such, we assessed the potential anti-hantaviral activity of 25-OHC. The addition of 25-OHC inhibited the replication of the virus in the infected cells (Fig 5E). These results suggest that cholesterol supplementation likely promotes ANDV infection, which, taken together with the rest of this lipidomic analysis, may indicate the extensive metabolic perturbations in ANDV-infected cells due to cholesterol depletion and downregulation of the cholesterol synthesis pathway.

### Evaluating potential drug candidates against ANDV

Due to its known ability for human-to-human transmission, as well as our observation of higher virulence in a broader range of cell types, we sought to identify potential antiviral agents targeting ANDV. For this purpose, we utilized the known antiviral biologic IFN-β, as well as the STING pathway agonists diABZI and cAIMP, to establish a drug testing platform [73,74] (S4A Fig). Calu-3 cells were pre-treated with drug compounds 24 hours prior to ANDV infection (MOI 0.1), and cells were fixed at 48 hpi. RNA samples were then collected to assess viral load. As a negative control, we used the protease inhibitor compound GC376, which has been shown to be effective against viruses encoding proteases, such as SARS-CoV-2 [75]. We observed that both IFN-β and STING agonists effectively inhibited ANDV replication (S4A-S4C Fig). As expected, GC376 did not show inhibition against ANDV, which does not encode for a protease.

**Fig 6:**
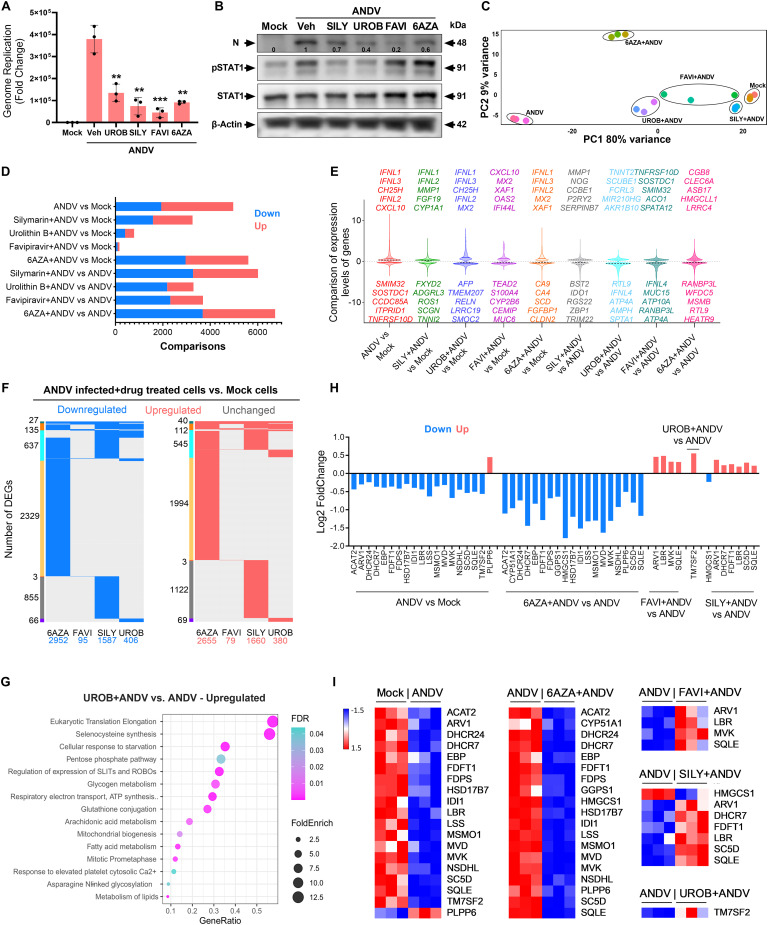
Pharmacogenomics Analysis of Compounds Exhibiting Anti-ANDV Activity in Lung Cells. **A)** The graph shows genome replication levels in indicated drug-treated ANDV-infected Calu-3 cells at 48 hpi. Dosages: UROB (urolithin B) and SILY (silymarin) = 30µM, FAVI (favipiravir) = 10µM, 6AZA (6-azauridine) = 5µM. Representative data presented from two experimental repeats. Student t-test was performed as compared with vehicle-treated ANDV-infected cells: **, P < 0.01; ***, P < 0.001. **B)** Western blot analysis of viral N protein levels in drug-treated infected cells. Levels of host IFN pathway transactivators phospho-STAT1 and STAT1 were also detected. Representative data presented from two experimental repeats. **C)** Principal-component analysis (PCA) plot shows global transcriptional response by drug-treated infected cells. **D)** The bar graph shows the total number of down- and up-regulated genes in indicated samples. **E)** Violin plot shows a comparison of expression levels (padj ≤ 0.01 with log2 Fold Change 1) of DEGs in various samples. **F)** Comparison of down- and up-regulated DEGs in drug-treated infected cells relative to uninfected cells. The sidebars show the DEGs group (green: DEGs shared by four datasets; orange: 3 datasets; turquoise: two datasets; others: exclusive to each dataset). **G)** Dot plot shows the up-regulated pathways in urolithin B-treated infected cells relative to ANDV infected cells. **H)** Bidirectional bar chart shows DEGs involved in cholesterol biosynthesis pathway among indicated drug-treated infected cells. **I)** Heatmaps show Z scores as expression levels of DEGs involved in the cholesterol biosynthesis pathway in uninfected (mock) and infected, as well as drug-treated infected, cells. Blue and red colors represent downregulated and upregulated genes, respectively.

Once this drug testing methodology was established, we subsequently tested the antiviral effectiveness of nucleoside analogs (favipiravir [76] and 6-azauridine [6AZA [77]]), as well as herbal compounds (silymarin [78] and urolithin [79]), which have been shown to possess antioxidant properties (S5 Table). We observed that these compounds imparted changes that significantly inhibited ANDV genome replication and infection at non-cytotoxic dose levels (Figs 6A-6B, S4D). To understand the molecular effects of these antiviral compounds, we utilized RNA-Seq methodologies to profile the transcriptomic changes. We found that host transcriptional response was differential in drug-treated cells, suggesting varying levels of ANDV inhibition in combination with the drug-specific host effect (Fig 6C-6E). This differential response was confirmed by the extreme coordinates on the principal-component analysis (PCA) plot for the drug conditions tested (Fig 6C). Cells treated with 6AZA and silymarin showed similar transcriptional response profiles, which varied from those seen in urolithin B- and favipiravir-treated cells when compared to vehicle-treated infected cells (Fig 6D-6E). There was significant overlap in DEGs across drug-treated infected cells, with the majority of the 799 commonly upregulated and 697 commonly downregulated genes being shared across two or more drug conditions when compared to uninfected healthy (mock) cells (Fig 6F). Among these DEGs, only 27 were downregulated and 40 were upregulated, with broad expression across the four drug-treated conditions. However, drug-treated infected cells had a range of 6–4,323 exclusive DEGs. In line with experimental results, it is noteworthy to observe that the least number of total and unique DEGs were identified in the urolithin B- and favipiravir-treated cells, confirming the higher efficiency of these two drugs in inhibiting ANDV (Figs 6D-6F, S4D). Host unique gene expression in nucleoside analog 6AZA-treated infected cells was increased 721- and 330-fold as compared to favipiravir- and urolithin B-treated infected cells, respectively. In the context of infection, this finding suggests that, in addition to inhibiting viral replication, 6AZA may also be involved in dysregulating transcriptional programming [80], which could manifest as adverse or detrimental effects at the molecular level.

Furthermore, we assessed the transcriptomic profiles of uninfected control cells treated with each of these four drugs or vehicle (DMSO). In this analysis, we did not observe any transcriptional differences at our standard statistical cutoff (padj 0.01, fold change 1) between drug-treated and vehicle-treated uninfected cells. To capture a broader range of transcriptional differences, we then analyzed the data using a less stringent cutoff (padj 0.01 without fold change constraints), which revealed 154 and 663 DEGs with low expression levels for silymarin- and 6AZA-treated uninfected cells, respectively (S5 Fig; S6 Table). Interestingly, no differentially expressed genes (DEGs) were identified for urolithin B or favipiravir, suggesting that these two drugs may have no effect on cellular transcription. Further exploring the pathways for DEGs in uninfected control cells treated with each of these drugs revealed that both silymarin and 6AZA upregulated the pathways involved in the regulation of cholesterol biosynthesis by SREBP, as well as activation of gene expression by SREBF (Sterol Regulatory Element Binding Transcription Factor) (S6 Table). Silymarin uniquely upregulated eukaryotic translation machinery, as well as the citric acid cycle and respiratory electron transport, whereas 6AZA upregulated transcriptional activation of mitochondrial biogenesis, circadian clock, and HATs acetylate histone, among other pathways. The nucleoside analog 6AZA specifically downregulated pathways involved in DNA synthesis, mitotic metaphase and anaphase, cellular response to hypoxia, and eukaryotic translation machinery (S6 Table). Silymarin downregulated pathways such as fibronectin matrix formation, laminin and syndecan interactions, and signaling by the TGF-β receptor complex. Taken together, this pharmacogenomics analysis of drug alone-treated cells suggests a possible mode of action for these drugs.

In further examining drug-treated infected cells, we utilized Pathway and Gene Ontology enrichment analyses, which showed upregulation of pathways involved in cell repair, cellular responses, and mitochondrial biogenesis and metabolisms, including glycogen metabolism, amino acids and derivates, metabolisms of glucose, fatty acid, carbohydrates, proteins, vitamins, and cofactors and lipids, as compared to vehicle-treated infected cells (Fig 6G, S5 Table). In contrast, 6AZA further downregulated most of these pathways compared to vehicle-treated infected cells. We observed that many immune, signaling, and cell death pathways, including apoptosis, death receptor signaling, necrosis, IFN signaling, NF-κB signaling, interleukin-10 signaling, and cytokine signaling, were commonly inhibited or downregulated in cells treated with any of the four drugs. This is potentially reflective of reduced viral replication because of drug treatment.

Importantly, ANDV infection of the vehicle-treated group downregulated many cell cycle and metabolic pathways, specifically those involved in lipid and cholesterol biosynthesis (Fig 5A). Except for 6AZA, the other three drugs were balanced or normalized with regard to the expression of several genes involved in the cholesterol synthesis pathway (Figs 6H-6I, S4E), suggesting that these drug-treated cells have retained cholesterol content for regular function. Expression levels of innate immune genes (IDO1, HELZ2) and inflammatory genes (IL6, ICAM1) were normalized upon drug treatment (S4E Fig). Taken together, our comprehensive pharmacogenomic analysis revealed distinct mechanisms of action for urolithin B, favipiravir, silymarin, and 6AZA, as well as pathophysiological molecular changes during ANDV infection.

## Discussion

The objective of this study was to establish biologically relevant, advanced human *in vitro* modeling systems to evaluate cell tropisms and host responses to infection with Old and New World hantaviruses. This is particularly necessary, given that existing biosafety level 4 (BSL-4) requirements currently prevent studies analyzing hantaviruses in rodent species that are highly permissive to hantaviral infection and capable of excreting the virus [81,82]. Because New World hantaviruses cause HCPS, we focused on utilizing various lung cell types, as well as heart cells, to investigate their effects on these cells. We found that the New World hantavirus ANDV readily infected a broad variety of cell types assessed – pulmonary endothelial cells, Calu-3 lung cells, hPSC-CMs, and hPSC-astrocytes. In contrast, we found that SNV, another New World hantavirus, exhibited robust infection only in lung endothelial cells. These findings support previous observations that HTNV, SNV, and ANDV can efficiently infect human endothelial cells [46,83–86]. Post-mortem analysis of SNV patients shows uniform infection of the lung endothelium [24]. HTNV is shown to proliferate in HUVECs [87], and ANDV readily infects microvascular endothelial cells [88] *in vitro*. Additionally, one investigation following 70 patients infected with PUUV, a New World hantavirus, found that 57% of patients showed abnormal cardiac findings, such as alterations in their ECGs, left ventricular contraction abnormalities, and mild pericardial effusion [89] – a finding that contrasts with the expected lack of cardiac involvement in HFRS. As such, there is a need for further detailed investigations into the ability of New World hantaviruses to infect cardiac cells and associated clinical presentations. While hantaviral involvement of the central nervous system is rare, *in vitro* studies have shown the capacity of hantaviruses to infect astrocytes, as we have shown, as well as other brain cells [59]. Clinical reports have showcased the abilities of SNV and ANDV, among other hantaviral species, to cause encephalitic symptoms and alterations in mental state [57,58,90–93].

More recent studies have begun to delineate key differences between Old and New World hantaviral cell entry mechanisms. SNV and ANDV glycoproteins have been shown to interact with Protocadherin-1 (PCDH1) to gain cell entry [22,94]. Additionally, two introduced point mutations in PCDH1 were demonstrated to protect Syrian hamsters from fatal ANDV infection [94]. However, PCDH1 was not required to facilitate cell entry of Old World hantaviruses into human lung endothelial cells, suggesting that these viral clades have evolved host and tissue tropisms independently [22,95]. Our systematic comparative study further highlights the differences between Old and New World hantaviral effects on various human cell types.

In hPSC-CMs and -astrocytes, while we did not observe much activation of innate immune pathways, pathways related to the cell cycle were readily upregulated in hPSC-CMs exposed to SNV. This observed effect is likely due to the virus not causing a robust infection in these cells. Like SNV, the Old World HTNV showed low levels of infection in Calu-3 cells. However, both SNV and HTNV induced the activation of IFN antiviral pathways in these lung cells, which is likely a cell-type-specific response aimed at restricting the further spread of infection. An independent study using a Syrian hamster model showed that pre-infection with SNV induced systemic antiviral immune responses, which is likely a generic response to restrict more widespread infection [96,97]. It is worth noting that we observed SNV to have a low level of infection in Calu-3 cells, although it did not establish active replication in hPSC-CM and hPSC-astrocytes. Therefore, the RNA-Seq analysis using total RNA provides a snapshot of the mean host response from both actively infected cells and cells carrying viral RNA, as well as bystander-uninfected cells. As such, further studies utilizing RNA-Seq analysis of separately sorted infected and uninfected cells, or single-cell RNA-Seq, may provide a more comprehensive picture of the host response to hantaviral infection.

The widespread and ready infection observed with ANDV across these various cell types suggests that the virus can suppress the antiviral response more efficiently. ANDV infection of pulmonary endothelial cells also showed strong upregulation of phospho-STAT1 on Western blot (Fig 1B), suggesting robust induction of type I IFN signaling and interferon-stimulated genes (ISGs). It has been posited that the ANDV N protein may restrict the induction of IFN-β and downstream ISGs [98]. This delay in IFN response may be the reason for rapid infection and more severe symptoms observed in ANDV and potentially other hantaviral infections. It is also known that pathogenic hantaviruses can inhibit the nuclear translocation of phospho-STAT, which can inhibit ISG responses in infected cells [99–105]. Other studies have shown that HTNV Gc and NP proteins can interact with tripartite motif protein 25 (TRIM25) to inhibit the type I IFN pathway by interfering with the interaction between TRIM25 and RIG-I/MDA-5 [106]. IFN production was also shown not to be inhibited in TRIM25-KO Sg25 hepatic cells, highlighting important interactions between HTNV NP/Gc proteins and the retinoic acid-induced gene I (RIG-I)-like receptor (RLR) pathways. In our study, we also found that TRIM25 is upregulated upon viral infection. HTNV has also been found to activate receptor-interacting protein kinase 3 (RIPK3), thereby inhibiting STAT1 phosphorylation and facilitating viral evasion of host immune response [107]. Utilizing an overexpression system for both SNV and ANDV, the Gc and NP proteins were found to antagonize the JAK/STAT pathway [108]. Though expression of both Gc and Np proteins is required for ANDV-driven inhibition of IFN production, SNV requires just Gc alone. Furthermore, ANDV NSs protein is able to interact with MAVS and suppress MDA5, RIG-I, or TBK1-mediated IFN-β promoter activity, although the exact mechanism of this interaction remains unknown [109]. A transcriptomics study found delayed activation of type I IFN response in ANDV-infected hamsters compared to HTNV infection [97]. It has also been observed that the RIG-I-like receptor (RLR) pathway was an essential pathway for anti-HTNV innate immune activation, IFN production, and ISG expression in human endothelial cells [110]. However, this expression was also dependent on viral replication levels. As such, there is a need for additional studies to better understand cellular immune responses to hantaviral infections, which can help in the development of effective vaccines.

In our transcriptomics and lipidomics studies of lung epithelial cells, we found that not only were all three viruses able to disrupt normal cell cycle and metabolic processes at the transcriptional level, but that ANDV-infected cells also had reduced levels of cholesterol biosynthesis genes, as well as cholesterol and triglycerides metabolites. In a previous siRNA screen of ANDV, it was found that the sterol regulatory pathway is necessary for viral entry [111]. It was also found that sterol response element binding factor-2 (SREBP2) was able to stimulate macrophages upon HTNV infection by promoting the production of inflammatory cytokines [112]. NEAT1–2 was also found to potentiate SREBP2 activity through the upregulation of *Srebf1*, promoting more inflammatory macrophage infiltration and limiting HTNV propagation, which showed a negative correlation between NEAT1–2 levels and HTNV-related HFRS progression. Additional studies have confirmed that cholesterol is required for cell entry by both ANDV [113] and HTNV [65], and that depletion of cellular cholesterol inhibits ANDV infection [114]. One study identified a specific need for high levels of cholesterol in the pathogenesis of both ANDV and HTNV [113]. Our observation of the reduction in cholesterol levels suggests that further investigation is necessary to better understand the viral interaction of ANDV with the cholesterol pathway. The observed downregulation may be a host antiviral response aimed at removing the cholesterol required for viral replication and spread. Other pandemic-causing and -potential viruses, including SARS-CoV-2 [115] and several types of flaviviruses [116–118], have also been observed to require high levels of cholesterol biosynthesis for viral replication and infection.

Our discovery of elevated LPC and LPE levels is consistent with similar phenomena observed during the course of SARS [119], SARS-CoV-2 [120], and Dengue virus [121] infections, with higher LPC and LPE levels linked to increased viral replication resulting from drastic alterations in cell membrane curvature and permeability. The downregulation of cellular cholesterol and the viral-mediated depletion of cholesterol that we observed may explain the disease outcomes associated with the virus. We propose that the depletion of cellular cholesterol can lead to increased cell membrane fragility, thereby increasing the likelihood of cell death and leakage, and potentially contributing to enhanced inflammation. Evidence of cell damage and necrosis has been reported upon infection with hantaviruses, particularly in the kidney and pituitary gland [122,123]. Taken together with our observation of ready PSC-cardiomyocyte infection, this dysregulation of cholesterol suggests that people with cardiovascular comorbidities may be at an elevated risk of more severe infection.

Our study revealed that STING agonists, nucleoside analogues, and plant-derived compounds exhibited antiviral activity. STING agonists are known to activate type I IFN antiviral response [74,124]. Silymarin and urolithin B likely promote an antiviral response by enhancing cellular metabolism and activating host antiviral pathways. Silymarin has demonstrated antiviral activity against hepatitis C virus, Dengue virus, Chikungunya virus, and HIV [78]. These host-directed antivirals can provide broad-spectrum protection against multiple families of viruses in the event of outbreaks. Host STING protein has been shown to promote IFN-independent antiviral pathways in response to HTNV infection, as assessed in HUVECs [125]. The nucleoside analog favipiravir’s activity against HTNV has been described previously [126], and we confirmed its therapeutic potential against ANDV. Favipiravir and urolithin B showed minimal transcriptomic changes upon treatment, which augurs well for their further development. Favipiravir has been shown to improve survival in ANDV- and SNV-infected Syrian hamsters [127]. Clinical trials have shown that humans can tolerate favipiravir with no significant adverse effects noted [127]. Favipiravir can directly bind to hantaviral RNA-dependent RNA polymerase (RdRp) coded by the L gene. This compound has also been approved for the treatment of influenza virus in Japan [128]. Thus, multiple RNA viral families share favipiravir inhibitory activity on RdRp, and a detailed Structure-Activity Relationships (SAR) study can yield additional information on its mode of action. Similarly, the direct activity of urolithin B on viral targets, if any, needs to be investigated. Despite exhibiting potent antiviral activity at non-cytotoxic doses, 6-AZA caused the largest changes in the transcriptional profile of treated cells. These unnecessary cellular changes can contribute to potential side effects. Thus, our pharmacogenomic analysis provided key safety details to inform decisions and advance compounds to the next phases of pre-clinical and clinical testing. Additional animal studies using Syrian hamsters are needed to characterize the *in vivo* antiviral activity of these promising drug candidates.

With the rapid evolution of RNA viruses observed in the past few years, it is becoming increasingly apparent that a strong pandemic preparedness infrastructure is essential. A key tenet of this preparedness is identifying these pandemic potential viruses and those most at risk. We expect that, in the event of an ANDV pandemic, those with lung and heart comorbidities will be at an increased risk of mortality due to the broad tropism of the virus. In this study, we have generated complex *in vitro* hPSC-derived model systems and transcriptomics data. Our results serve as important resources for the research community to further advance our collective understanding of ANDV and hantaviral cell tropisms and infection mechanisms. We also provide valuable information for drug evaluation and a reliable framework to analyze compounds that can be further developed and potentially utilized in the event of an ANDV pandemic. Additional future work will focus on understanding viral tropism to the nasal epithelium and upper respiratory tract using air-liquid interface (ALI) culture models, as well as delineating the interaction of hantaviruses with immune cells. Moreover, analyzing the cellular signaling pathways in response to the virus in both endothelial cells and immune cells may enhance our understanding of virus pathogenic mechanisms and aid in identifying novel therapeutic targets. Taken together, broad and multifaceted antiviral strategies are crucial for addressing the dynamic challenges posed by these emerging viral threats.

### Study limitations

This study does have some limitations. First, the viral strains used were isolated from rodent hosts. Clinical isolates from humans would provide additional insights into host responses and viral replication. Obtaining these isolates from infected individuals has been difficult, however, due to the infrequent occurrence of infection and/or the timing of diagnosis, which may be after viral clearance or mortality [129]. Developing reverse genetics systems can facilitate the synthesis or resurrection of hantaviruses based on complete viral sequences available from human infections. Additionally, we acknowledge that there is an inherent limitation of transcriptomic studies, as the tested viruses replicated at varying levels in lung epithelial cells, hPSC-CMs, and hPSC-astrocytes. Therefore, we are unable to make an equal comparison of gene expression when viral RNA levels are similar. Using a bulk RNA sequencing approach, the transcriptional responses of both infected and uninfected bystander cells were averaged, a method that can be improved by employing single-cell RNA sequencing or by enriching for infected and uninfected cells separately. For this study, we used an antibody generated against SNV to detect all three viruses. We acknowledge that the antibody may be less sensitive against HTNV epitopes, as evidenced by the lower level of positive cells observed through IFA compared to the high level of viral genome detected via RT-qPCR. The data presented are primarily from *in vitro* cell culture systems, as BSL-4 containment is required to conduct *in vivo* animal studies using these viruses. However, we have utilized a more advanced and biologically relevant *in vitro* human 3D organoid system, which can serve as a valuable tool in drug development.

## Methods and materials

### Ethics statement

This study was performed in strict accordance with the recommendations of UCLA. All ANDV, SNV, and HTNV live virus experiments were performed at the UCLA BSL-3 High Containment facility. Human lung tissue was obtained from deceased tissue donors in compliance with consent procedures developed by the International Institute for the Advancement of Medicine and approved by the Cedars-Sinai Medical Center Internal Review Board. The pluripotent stem cell studies were approved by the UCLA and City of Hope IRB Stem Cell Oversight Committees.

#### Cells.

Primary Human Pulmonary Artery Endothelial Cells (HPAEC) were obtained from the American Type Culture Collection (ATCC, PCS-100–022) and cultured in Vascular Cell Basal Media (PCS-100–030, ATCC), supplemented with Endothelial Cell Growth Kit-VEGF (PCS-100–041) and Penicillin-Streptomycin-Amphotericin B Solution (PCS-999–002, ATCC). The cells were maintained in a humidified environment at 37°C, 5% CO_2_, and subcultured following the manufacturer’s recommended specifications. Primary Human Pulmonary Endothelial Cells were used for experiments within passages 2–5. Human lung adenocarcinoma epithelial cells (Calu-3) were purchased from the ATCC (ATCCHTB-55). These were cultured in Eagle’s Minimum Essential Medium (EMEM) (Corning), supplemented with 20% fetal bovine serum (FBS),1% MEM Non-Essential Amino Acids Solution (MEM NEAA), 1% L-glutamine (L-glu) and 1% penicillin/streptomycin (P/S). These Calu-3 cells were incubated at 37°C with 5% CO_2_. Human induced pluripotent stem cell (hiPSC) differentiation into astrocytes was induced using 0.1 μM retinoic acid (RA), 4 μM CHIR99021, 3 μM SB431542, and 2 μM Dorsomorphin for 2 days, followed by continued induction for 5 days with the removal of Dorsomorphin [130,131]. Neural progenitor cells (NPCs) were derived from hiPSCs and further differentiated by treating them with 10μM retinoic acid (RA) and the smoothed agonist (SAG) for 5 days. The NPC spheres were dissociated into single cells using accutase, then plated into Matrigel-coated Growth Factor Reduced (GFR) Basement Membrane Matrix, LDEV-free (from Corning), plates at a density of 1 × 10^5^ cells per well in 6-well plates. To generate astrocyte precursors, cells were cultured for 10 days in N2B27 medium (DMEM/F12, 1xN2, 1xB27, 1xNEAA, 1xGlutamax) plus 0.1 μM RA and 1 μM SAG. They were then switched to PDGF medium (1X N2, 1XB27, 10 ng/ml PDGFAA, 5 ng/ml HGF, 10 ng/ml IGF-1, 10 ng/ml NT3, 100 ng/ml Biotin, 60 ng/ml T3, 1 μM cAMP and 25 μg/ml insulin) for another 20 days. Astrocyte maturation was continued from NPC-derived astrocyte precursors without passaging. To mature the astrocytes, the cells were switched into astrocyte maturation medium (DMEM/F12, 1xN2, 1xB27, 1xNEAA, 2 mM GlutaMAX, and 10 ng/ml CNTF) for another 7 days. Human pluripotent stem cell-derived cardiomyocytes (hPSC-CMs) were provided by the UCLA Cardiomyocyte Core and were derived as described below. The hPSC-CMs were differentiated from human embryonic stem cell (hESC) line H9. The hPSCs were maintained in mTeSR1 (STEMCELL Technology), and RPMI1640 [supplemented with B27 minus insulin (Invitrogen)] was used as a differentiation medium. From Days 0–1, 6 μM CHIR99021 was added into the differentiation medium. On Days 3–5, 5 μM IWR1 (Sigma-Aldrich) was added to the differentiation medium. Thereafter, on Day 7, RPMI 1640 plus B27 maintenance medium was added. Finally, on Days 10–11, RPMI 1640 medium without D-glucose and supplemented with B27 was used for the transient metabolic purification of CMs. All cell types used were confirmed to be free of mycoplasma contamination.

#### Human distal lung organoid studies.

The culture of human distal lung epithelial organoids was performed following previously established protocols in our laboratory [54,132]. In summary, a mixture of 5,000 fluorescence-activated cell sorting (FACS)-enriched distal lung epithelial cells and 7.5x10^4^ MRC5 human lung fibroblast cells (ATCC CCL-171) were prepared in a 50:50 (v/v) solution of ice-cold Matrigel [Growth Factor Reduced (GFR) Basement Membrane Matrix, LDEV-free from Corning] and PneumaCult ALI media. 100 μL of this suspension was seeded onto the apical surface of a 0.4μm pore-size cell culture insert within a 24-well-supported format. Following Matrigel polymerization, 700 μL of PneumaCult ALI media was added to the basement membrane. The media was then supplemented with 50 μg/mL of Gentamycin (Sigma Aldrich) for the first 24 hours and 10 μM Rho kinase inhibitor for the first 48 hours. Subsequently, 2 μM CHIR-99021 (STEMCELL Technologies), a Wnt pathway activator, was added to the media at 48 hours and maintained through the culture period. Media was replaced every 48 hours, with cultures maintained at 37^o^C in a humidified incubator (5% CO_2_). After 15–20 days, the organoids were subjected to hantaviral infection (MOI 0.1). For MOI calculations, the organoids were single-cell dissociated to obtain the total cell number per well using accutase from three extra wells of organoids. At 48 hpi, the organoids were either lysed in Trizol for RNA isolation or fixed with 4% paraformaldehyde (PFA) for 30 minutes for IFA analysis.

#### Viruses.

ANDV (Chile-9717869 strain) and SNV (SNV-77734) were obtained from Dr. Heinz Feldmann from the NIH/NIAID, and HTNV (Fojnica strain) was obtained from BEI Resources (S7 Table). The ANDV Chile-9717869 strain was provided by Dr. Connie Schmaljohn, U.S. Army Medical Research Institute of Infectious Diseases, Ft. Detrick, MD, to Dr. Heinz Feldmann. The Chile-9717869 strain of ANDV was first isolated from an infected *Oligoryzomys longicaudatus* rodent in 1997 [133]. The SNV-77734 strain was provided to Dr. Feldmann by Dr. Brian Hjelle, University of New Mexico Health Sciences Center (HSC), Albuquerque, NM. The SNV-77734 strain was originally isolated from a single wild deer mouse (*Peromyscus maniculatus rufinus*) in 2000 [134]. The HTNV Fojnica strain was isolated in *Apodemus flavicollis* in the Fojnica region of former Yugoslavia (now Bosnia and Herzegovina) in 1989 [135]. We have amplified these viruses once in Vero E6 cells. Viral stocks were made from cell-free supernatants collected at Day 6 post-infection, aliquoted, and stored at -80°C. We verified the sequences of the S and M genomic segments and confirmed their sequence to respective strains. The virus titer was measured in Vero E6 cells with the established 50% tissue culture infectious dose (TCID50) assay.

#### Viral infection.

Human pulmonary endothelial cells, lung epithelial cell line (Calu-3), hPSC-astrocytes, and hPSC-CMs were plated at 1x10^5^ cells per well using a 48-well plate. Viral inoculum of ANDV (Chile-9717869 strain), HTNV (Fojnica strain), and SNV (SNV-77734) was added onto the cells at a multiplicity of infection (MOI) of 0.01 or 0.1 using serum-free base media. After 1 hour of incubation at 37°C with 5% CO_2_, the inoculum was replaced with respective cell-type media. Cells were then fixed at selected time points with methanol (incubated in -20°C freezer for 20 minutes followed by PBS wash) or 4% PFA for 30 minutes at room temperature and subsequently washed three times with ice-cold PBS. For protein analysis, cells were lysed using ice-cold RIPA buffer (60 µL/well of 48-well plate) for 3 minutes. The protein lysates were harvested and briefly vortexed before storing at -80°C. For RNA samples, the cells were lysed using Trizol for 5 minutes. Vials containing Trizol lysates were stored at -80°C prior to RNA isolation. For long-term Calu-3 infection studies, half-media change was performed in both infected and mock cell wells every other day.

#### Viral titer by TCID50 (*Median tissue culture infectious dose*) assay.

Viral production by infected cells was quantified by the TCID50 assay, as previously described with modifications [48]. Vero E6 cells (density of 5 x10^3^ cells/well) were plated in 96-well plates. The next day, viral culture media were serially diluted 10-fold (10^1^ to 10^8^) and added to Vero E6 cells. These cells were incubated at 37°C with 5% CO_2_. 3–4 days later, the cells were lysed in Trizol for virus-specific RT-qPCR or fixed with 4% PFA for 30 minutes and subjected to immunostaining with mouse (serum) polyclonal anti-Sin Nombre Virus SN77734 Nucleocapsid Protein Antibody (BEI Resources CAT# NR-9676) (S7 Table). This antibody is cross-reactive with nucleocapsid proteins of both ANDV and HTNV. The wells positive for viral infection were identified for each dilution. Then, the dilutions immediately above and below 50% of viral inhibition were determined. TCID50 was calculated based on the method of Reed and Muench.

#### Drug compounds and infections.

The compounds tested were obtained from InvivoGen, Millipore Sigma, and Selleckchem (S7 Table). All compounds were provided as lyophilized and were then reconstituted in Nuclease-Free water (Invitrogen) or DMSO. Compounds were then aliquoted and stored at either -80°C or room temperature in dry conditions. For drug studies, indicated drugs were added in Calu-3 cells 24 hours prior to ANDV infection (MOI 0.1). At 48 hpi, protein and RNA samples were collected for Western blot and RT-qPCR analysis. In cholesterol-related studies, Calu-3 cells were treated with cholesterol or 25-OHC compounds 2 hours prior to ANDV infection. The cells were then fixed with 4% PFA for 30 minutes at 48 hpi for IFA analysis.

#### Immunohistochemistry.

Cells were fixed with methanol (incubated in -20°C freezer until washed with PBS) or 4% PFA for 30 minutes. The cells were washed three times with 1x PBS and permeabilized by incubating in blocking buffer (0.3% Triton X-100, 2% BSA, 5% Goat Serum, 5% Donkey Serum in 1X PBS) for 1 hour at room temperature. For immunostaining, cells were incubated overnight at 4°C with each primary antibody, then washed with 1X PBS three times and incubated with respective secondary antibody [Goat anti-Mouse IgG (H + L) Cross-Adsorbed Secondary Antibody, Alexa Fluor 555 (Thermo Fisher Scientific, CAT#A-21422); Goat anti-Rabbit IgG (H + L) Cross-Adsorbed Secondary Antibody, Alex Fluor 488 (Thermo Fisher Scientific, CAT#A-11008] (S7 Table) for 1 hour at room temperature. The cell nuclei were stained with DAPI (4’,6-Diamidino-2-Phenylindole, Dihydrochloride) (Life Technologies) at a dilution of 1:5000 in 1X PBS. Image acquisition was done using Leica DM IRB fluorescent microscopes.

#### Image analysis/quantification.

Microscope two-dimensional images were obtained using the Leica DM IL LED Fluo and Leica LAS X Software Program. 2–3 two-dimensional images were captured per well at 48 hpi for each condition. These images were quantified using Image J’s plugin (Multipoint and Cell Counter). The positively stained cells were counted by a double-blinded approach. Confocal slide samples were imaged using a Leica SP8 MP-DIVE-FLIM Microscope at the Advanced Light Microscopy/Spectroscopy Laboratory and Leica Microsystems Center of Excellence at the California NanoSystems Institute at UCLA (RRID: SCR_022789) with funding support from NIH Shared Instrumentation Grant S10OD025017 and NSF Major Research Instrumentation grant CHE-0722519. Confocal three-dimensional images were collected in 1024x1024 format using a 63x oil immersion objective lens, fixed scan rate of 8000Hz, and averaged 12 times. Excitation laser lines and emission detection wavelengths were optimized for the fluorescent tags as follows: blue channel excitation of 405nm with emission detection range of 420–470nm, green channel excitation of 488nm with emission detection range of 500nm-530nm, and red channel excitation of 552nm with emission detection range 590–650nm.

#### Lipidomics.

Cells collected from three biological replicates per condition were transferred to extraction tubes containing phosphate-buffered saline (PBS). Subsequently, a modified Bligh and Dyer extraction method [136] was employed to process the samples. Prior to the biphasic extraction step, a mixture of 70 lipid standards across 17 subclasses (AB Sciex 5040156, Avanti 330827, Avanti 330830, Avanti 330828, Avanti 791642) was added to each sample as an internal standard. Following two consecutive extractions, the pooled organic layers were dried down in Thermo SpeedVac SPD300DDA using ramp setting four at 35°C for 45 minutes with a total run time of 90 minutes. The dried lipid samples were then resuspended in a 1:1 methanol/dichloromethane solution with 10 mM ammonium acetate and transferred to RoboVials (Thermo 10800107) for subsequent analysis. The samples were analyzed by direct infusion on a Sciex 5500 instrument equipped with a Differential Mobility Device (DMS) (comparable to the Sciex Lipidyzer platform). A targeted acquisition list consisting of 1,450 lipid species across 17 subclasses was used. The DMS was tuned with EquiSPLASH LIPIDOMIX (Avanti 330731). Data analysis was performed with an in-house data analysis workflow. Detailed information regarding instrument settings, multiple reaction monitoring (MRM) lists, and analysis methods are available [137,138]. Quantitative values were normalized to cell counts.

#### Phylogeny.

For the phylogenetic analysis, all 68 M segments of viral sequences from *Hantaviridae, Phenuiviridae, Nairoviridae, Arenaviridae*, and *Peribunyaviridae* families of the *Elliovirales* and *Hareavirales* orders (S1 Table) were aligned using MAFFT v.7.505 [139] and subsequently, these aligned sequences were used to identify GTR + F + R6 as a best-fit model based on the Bayesian Information Criteria using ModelFinder [140]. The phylogenetic tree was constructed using the maximum-likelihood (ML) method with 1,000 bootstrap replicates in IQ-TREE multi-core version 2.0.3 [141]. The phylogenetic tree was annotated in Interactive Tree Of Life (iTOL) [142].

#### RNA sample preparation and RT-qPCR analysis.

RNA was extracted from various virus-infected and drug-treated cell types using the RNA Mini Kit (BioRad) according to the manufacturer’s guidelines. Quantification of RNA was performed with a NanoDrop 2000 Spectrophotometer (Thermo Fisher Scientific). For cDNA synthesis, 1 µg of RNA was used alongside random hexamer primers and the SuperScript III reverse transcriptase kit (Thermo Fisher Scientific). qPCR was then carried out using either SYBR Green ROX Supermix (Life Technologies) on an Applied Biosystems QuantStudio 12K Flex RT-PCR system (Thermo Fisher Scientific) or SSOAdvanced Universal SYBR Green Supermix (Bio-Rad) with a CFX384 Touch RT-PCR detection system (Bio-Rad). The reactions were conducted in 10 µL volumes within a 384-well plate, with thermal cycling conditions starting with an initial denaturation at 95°C for 30 seconds, followed by 40 cycles of 95°C for 15 seconds and 60°C for 60 seconds. A melt-curve analysis was performed from 65°C to 95°C, with increments of 0.5°C every 2–5 seconds. The 2-ΔCT method was used for transcript quantification, normalizing against glyceraldehyde-3-phosphate dehydrogenase (GAPDH) CT values. Baseline mRNA levels in mock-treated cells were set to 1, and fold changes in infected cells were calculated relative to this baseline. Details of qPCR primer sequences for the target mRNA transcripts can be found in S7 Table.

#### RNA sequencing and data analysis.

Total RNA samples were prepared as described above. For every treatment condition, duplicate (quadruplicate samples pooled separately as duplicates) or triplicate RNA samples were submitted to the UCLA Technology Center for Genomics & Bioinformatics (TCGB) for RNA sequencing analysis. Library preparation, sequencing, and RNA-Seq data analysis were performed as described [74,143] with minor modifications. In summary, libraries were prepared with the KAPA Stranded mRNA-Seq Kit, followed by second strand synthesis converting the cDNA:RNA hybrid to double-stranded cDNA (dscDNA) and incorporating dUTP into the second cDNA strand. cDNA generation was followed by end repair to generate blunt ends, A-tailing, adaptor ligation, and PCR amplification. Different adaptors were used for multiplexing samples in one lane. Sequencing was performed on Illumina Novaseq 6000 for a paired-end 2x50 bp run. Data quality checking was done on Illumina SAV. Demultiplexing was performed with Illumina Bcl2fastq v2.19.1.403 software. Partek Flow [49] was used for all data analysis. Illumina reads from all samples were aligned to the human GRCh38 reference genome using STAR 2.7.9a [50,51], and Ensemble transcripts release GRCh38.107 GTF was used for gene feature annotation. Subsequently, the read counts per gene were qualified.

The differential gene expression analysis was performed using DESeq2 v1.40.1 in R v4.3.0 [52]. The median of ratios method was used to normalize expression counts for each gene across all samples studied. DEGs were considered if they were supported by a false discovery rate (FDR) *p* < 0.01 or pad 0.01 & FC 1. Unsupervised principal component analysis (PCA) was performed using DESeq2 in R v4.3.0. Reactome pathway analysis was performed using human all genes as the reference dataset in the Reactome v84 [55] of PANTHER v17.0-19.0 [53]. Reactome pathways were only considered if they were supported by FDR P < 0.05. The ggplot2 v3.4.2 in R and Prism GraphPad v9.5.1 were used to generate figures. The heatmaps were generated using pheatmap v1.0.12 in R. We deposited RNA-seq data to the NCBI GEO under the accession number GSE232641.

#### Western blot analysis.

For protein analysis, cells were lysed in ice-cold RIPA buffer (50 mM Tris pH 7.4, 1% NP-40, 0.25% sodium deoxycholate, 1 mM EDTA, 150 mM NaCl, 1 mM Na3VO4, 20 Mm or NaF, 1mM PMSF, 2 mg ml^-1^ aprotinin, 2 mg ml^-1^ leupeptin and 0.7 mg ml^-1^ pepstatin) or Laemmli Sample Buffer (Bio-Rad, Hercules, CA) for 3 minutes. These protein lysates were heated to 95°C for 5 minutes, and the samples were then resolved by SDS-PAGE using 10% gradient gels (Bio-Rad). They were subsequently transferred to a 0.2 µm PVDF membrane (Bio-Rad). After the transfer, the membranes were blocked (5% skim milk and 0.1% Tween-20) in 1x TBST (0.1% Tween-20) at room temperature (RT) for 1 hour. The membranes were then incubated with the respective primary antibodies [mouse (serum) polyclonal anti-Sin Nombre Virus SN77734 Nucleocapsid Protein Antibody (BEI Resources CAT#NR-9676); Sin Nombre Virus Glycoprotein 1 Antibody – BSA Free (Novus Biologicals, CAT#NBP2-41255-0.025mg); Phospho-Stat1 (Tyr701) (58D6) Rabbit mAb (Cell Signaling, CAT#9167S); Stat1 (D1K6Y) Rabbit mAb (Cell Signaling, CAT#14994S); Monoclonal Anti-Beta-Actin, Clone AC-74 produced in mouse (Millipore Sigma, CAT#A2228)] (S7 Table) overnight at 4°C subsequent to wash five times with 1% TBST. Respective secondary antibody conjugated to HRP [Anti-mouse IgG, HRP-linked Antibody (Cell Signaling, CAT#7076P2); Anti-rabbit IgG, HRP-linked Antibody (Cell Signaling, CAT#7074P2)] (S7 Table) and detected by SuperSignal West Femto Maximum Sensitivity Substrate (Thermo Scientific). Membranes were exposed and visualized with the Bio-Rad ChemiDoc MP Imaging System.

#### Statistics and data analysis.

GraphPad Prism, version 9.5.1, was used for graph generation and statistical analysis. Data was then analyzed for statistical significance using an unpaired student’s *t*-test to compare two groups (uninfected vs. infected) or a non-parametric t-test (Mann-Whitney Test). All data is representative of 2 or more experiments with 3–4 biological replicates. All statistical testing was performed at the two-sided alpha level of 0.05.

## Supporting information

S1 Fig Evaluating cell tropism of various hantaviruses. **A)** Phylogenetic analysis of aligned and sequenced M segment of viral sequences (n = 68) from *Hantaviridae, Phenuiviridae, Nairoviridae, Arenaviridae,* and *Peribunyaviridae* families of the *Bunyavirales* order. In the *Hantaviridae* cluster: SNV = circle; ANDV = square; and HTNV = star. The adjacent diagram presents the transmission modes of these three viruses. Image created on BioRender. **B)** The graphs demonstrate the differing levels of viral genome replication of hantaviruses in Vero E6 cells. **C)** The graph shows the count of hPSC-CM beats in Mock and ANDV-infected cells at 48hpi. **D)** Graphs represent the relative expression of various immune genes in Calu-3 cells and hPSC-CMs. Quantitative data are presented as mean ± standard deviation. Statistical comparisons were made using ANOVA followed by Tukey’s post hoc test (*, P < 0.05; **, P < 0.001).
(TIF)

S2 Fig Transcriptomic analysis of hantaviral infection in hPSC-CMs. **A, B)** The dot plot shows the pathway analysis of ANDV-infected/mock-infected and ANDV-infected/HTNV-infected hPSC-CMs at 48 hpi. **C, D)** Heatmap illustrates Z scores as expression levels of the genes involved in the indicated pathways in mock and infected hPSC-CMs. Red and green represent upregulated and downregulated genes, respectively. The corresponding volcano plots illustrate the differential expression of statistically significant genes of these pathways.
(TIF)

S3 Fig Examining hantavirus-induced dysregulation of cholesterol biosynthesis and metabolism pathways. **A)** The dot plot depicts the impact of ANDV on the expression of cholesterol pathway-associated genes and other indicated cellular pathways in hPSC-astrocytes. **B)** Heatmap illustrating Z scores representing reduced expression levels of genes associated with the cholesterol biosynthesis pathway. Red and blue correspond to up- and down-regulation, respectively. **C)** Bar graphs display the class average of lysophosphatidylethanolamines (LPE, left) and lysophosphatidylcholines (LPC, right) in Calu-3 cells infected with SNV (blue), ANDV (red), or mock (gray) at 24- and 48-hours post-infection (hpi). **D)** The graphs show the triglyceride (TG) bonds and TG carbon levels present in the mock and infected Calu-3 cells at 24 and 48 hpi. Quantitative data are presented as mean ± standard deviation. Statistical analysis was performed using ANOVA, followed by Tukey’s post hoc test *, P < 0.05; **, P < 0.01; ***, P < 0.001).
(TIF)

S4 Fig Pharmacogenomic analysis of potential anti-ANDV drug compounds. **A)** Immunofluorescence images of ANDV-infected Calu-3 cells treated with vehicle or various drug compounds at 24 hpi. Red = N protein. Scale bar = 25μm. **B)** The graph shows the levels of viral genome replication at 24 hpi in response to indicated drug compounds. **C)** The graph represents relative gene expression levels of the innate immune gene OAS1 in response to treatment with indicated drug compounds. **D)** The graphs illustrate the dose-response viability assay of indicated drug compounds in Calu-3 cells at 48 hours post-treatment. **E)** The bar charts show the varying Log2(Fold Change) values of indicated genes in ANDV-infected, as well as drug-treated infected, cells at 48hpi. Quantitative data are presented as mean ± standard deviation. Statistical analysis was performed using ANOVA, followed by Tukey’s post hoc test (*, P < 0.05; **, P < 0.001). ANDV = Andes virus; OAS1 = oligoadenylate synthetase 1; ANOVA = analysis of variance.
(TIF)

S5 Fig Transcriptomic profile of uninfected control Calu-3 cells treated with indicated antivirals. **A)** Bar chart shows the number of downregulated (blue) and upregulated (red) DEGs in Calu-3 control cells treated with Urolithin B (UROB), Silymarin (SILY), Favipiravir (FAVI), or 6-Azauridine (6AZA) at 48 hours post-drug alone treatment compared to vehicle-treated (Mock) cells. Asterisks indicate conditions with no significant DEGs. **B)** Violin plot shows patterns of differential gene expression levels in Calu-3 control cells upon drug treatments. The 5 most down- or up-regulated DEGs (padj < 0.01) were displayed. Asterisks indicate conditions with no significant DEGs. **C)** Venn diagrams illustrate the number of common and distinct genes downregulated (left) and upregulated (right) in Calu3 control cells treated with SILY (pink) or 6AZA (cyan) compared to mock. **D, E)** Dot plots represent most overrepresented Reactome pathways among downregulated (left) and upregulated (right) genes following treatment with SILY or 6AZA, compared to mock.
(TIFF)

S1 Table *Bunyavirales* order viruses used for ML phylogeny. Details of M gene sequences (n = 68) of various viruses belonging to the *Bunyavirales* order used from NCBI to construct the ML phylogeny (Related to Fig 1A). Each row is color-coded to match the phylogenetic cluster colors in Fig 1A.
(XLSX)

S2 Table Differentially enriched reactome pathways in hantavirus-infected pulmonary endothelial cells. List of significantly upregulated and downregulated Reactome pathways (Padj < 0.01; Fold Change ≥ 1) identified in pulmonary endothelial cells infected with three hantaviruses (ANDV, SNV, and HTNV) at 48 hpi (Related to Fig 1H-J).
(XLSX)

S3 Table Commonly upregulated genes in various cell types at 48 hpi. List of all the commonly upregulated gene counts in ANDV-, SNV- and HTNV-infected cell types at 48 hpi (Related to Fig 4).
(XLSX)

S4 Table Commonly up- and downregulated hantaviral cell entry receptors in infected cell types at 48 hpi. List of all the commonly up- and down-regulated putative hantaviral cell entry receptor genes in ANDV-, SNV- and HTNV-infected cell types at 48 hpi.
(XLSX)

S5 Table Hantaviral annotation of DEG sets in each cell type and drug treatment using PANTHER and overrepresented downregulated reactome pathways. Hantaviral annotation of DEG sets in each cell type and drug treatment performed using PANTHER, as well as the list of overrepresented downregulated Reactome Pathways, are presented (Related to Fig 5).
(XLSX)

S6 Table Differentially enriched reactome pathways in uninfected drug-treated Calu-3 control cells. List of significantly upregulated and downregulated Reactome pathways (Padj < 0.01) identified in Calu-3 lung epithelial control cells treated with antiviral drugs Silymarin [SILY] and 6-Azauridine [6AZA]) at 48 hpi (Related to S5D, S5E Fig).
(XLSX)

S7 Table Reagents and resources used in this study. (XLSX)
