# Supplementary figures and images for: Differential tropisms of old and new world hantaviruses influence virulence and developing host-directed antiviral candidates
Source: PLoS Pathog. 2025 Aug 26;21(8):e1013401. doi: 10.1371/journal.ppat.1013401 (PMC12380290; doi:10.1371/journal.ppat.1013401)

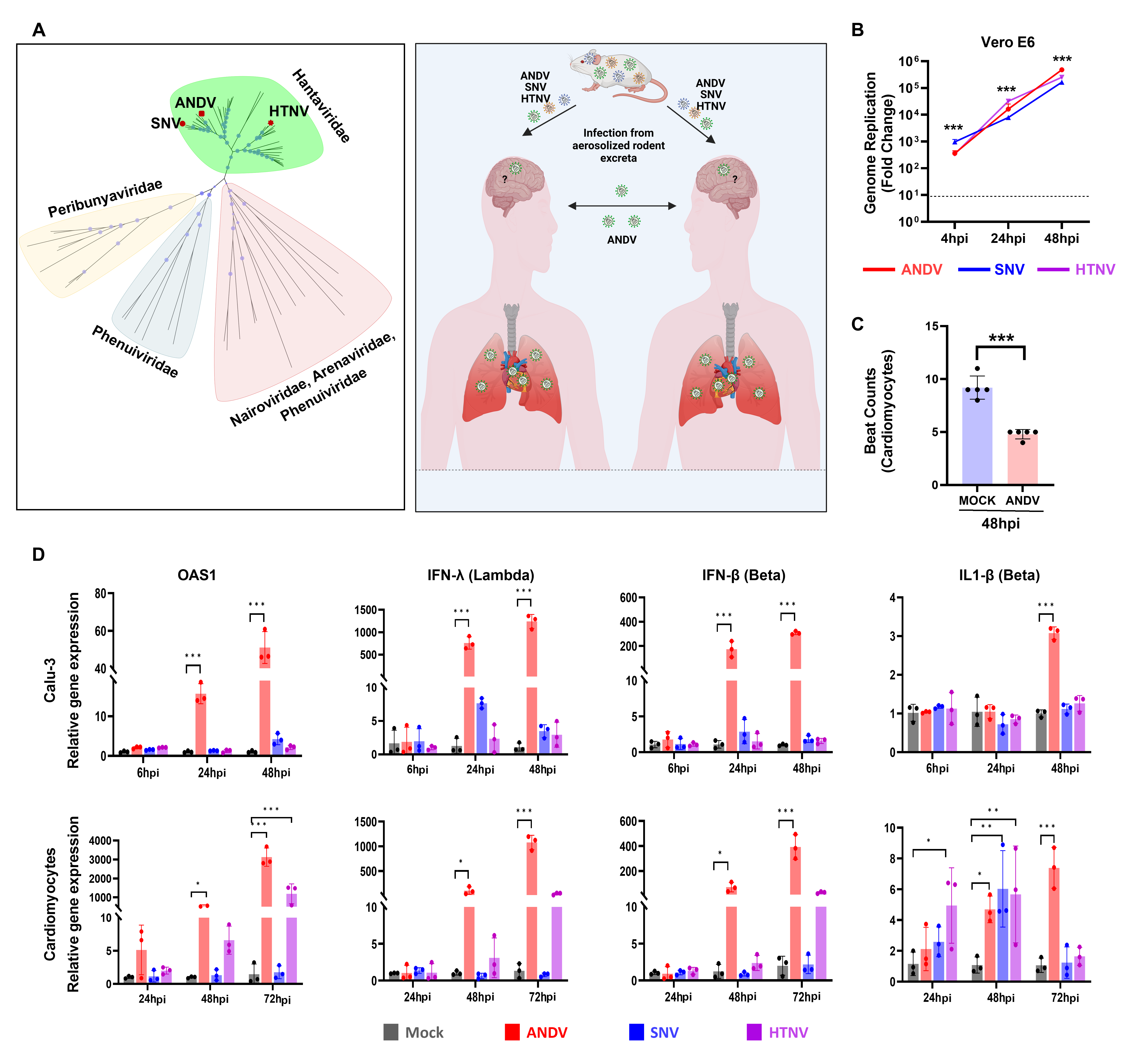

Supplement: S1 Fig — A) Phylogenetic analysis of aligned and sequenced M segment of viral sequences (n = 68) from Hantaviridae, Phenuiviridae, Nairoviridae, Arenaviridae, and Peribunyaviridae families of the Bunyavirales order. In the Hantaviridae cluster: SNV = circle; ANDV = square; and HTNV = star. The adjacent diagram presents the transmission modes of these three viruses. Image created on BioRender. B) The graphs demonstrate the differing levels of viral genome replication of hantaviruses in Vero E6 cells. C) The graph shows the count of hPSC-CM beats in Mock and ANDV-infected cells at 48hpi. D) Graphs represent the relative expression of various immune genes in Calu-3 cells and hPSC-CMs. Quantitative data are presented as mean ± standard deviation. Statistical comparisons were made using ANOVA followed by Tukey’s post hoc test (*, P < 0.05; **, P < 0.001). (TIF) [file ppat.1013401.s001.tif]

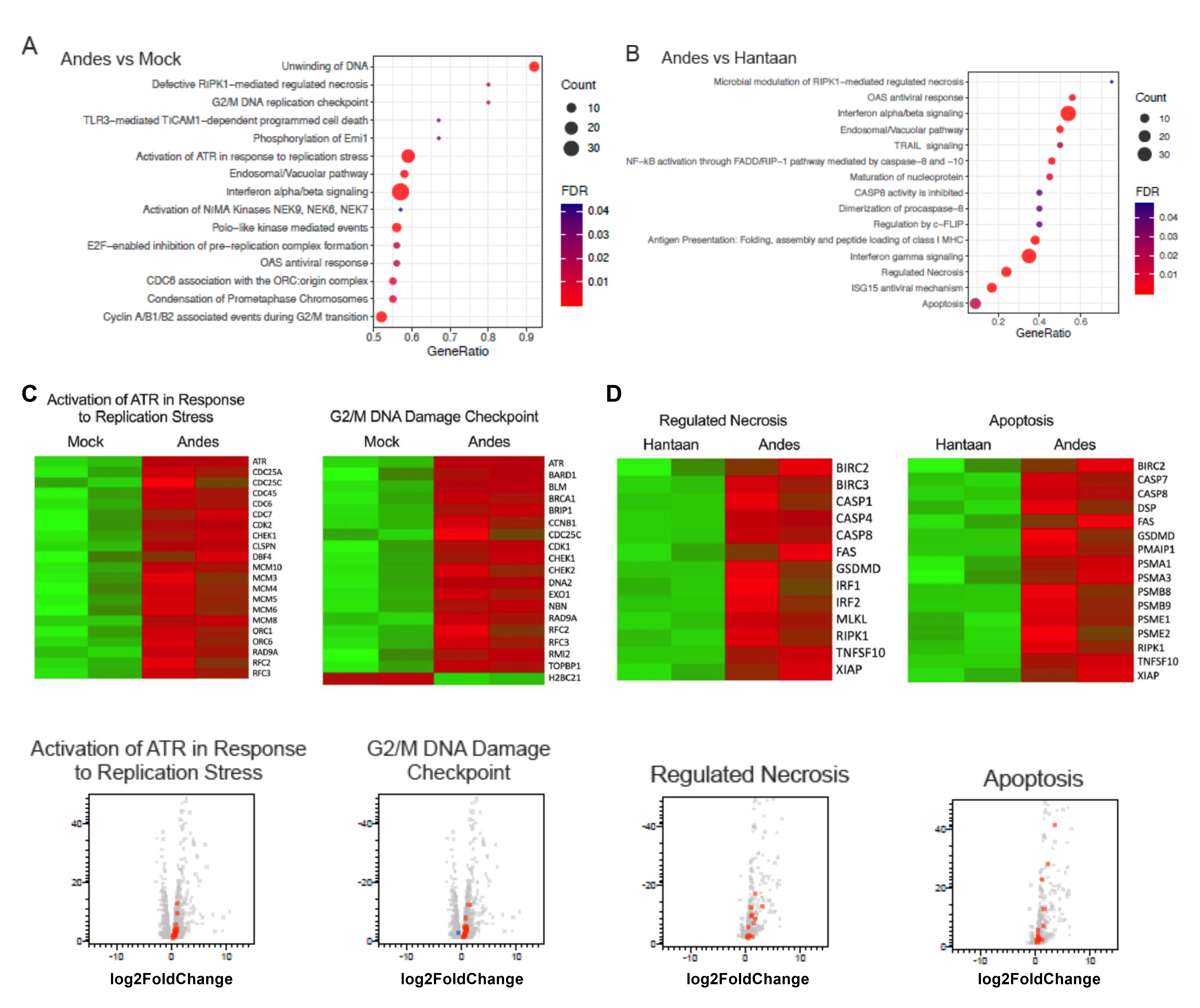

Supplement: S2 Fig — A, B) The dot plot shows the pathway analysis of ANDV-infected/mock-infected and ANDV-infected/HTNV-infected hPSC-CMs at 48 hpi. C, D) Heatmap illustrates Z scores as expression levels of the genes involved in the indicated pathways in mock and infected hPSC-CMs. Red and green represent upregulated and downregulated genes, respectively. The corresponding volcano plots illustrate the differential expression of statistically significant genes of these pathways. (TIF) [file ppat.1013401.s002.tif]

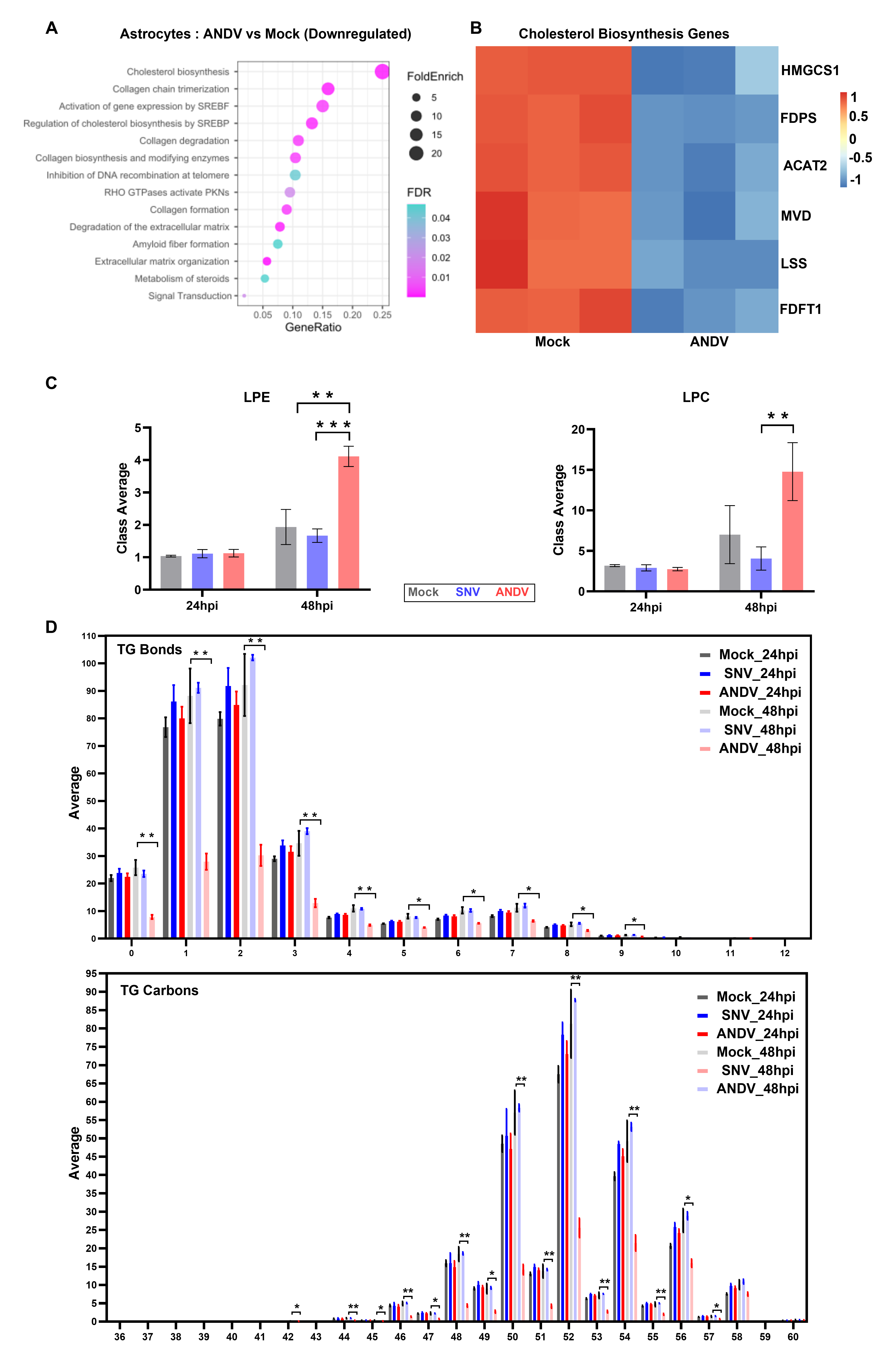

Supplement: S3 Fig — A) The dot plot depicts the impact of ANDV on the expression of cholesterol pathway-associated genes and other indicated cellular pathways in hPSC-astrocytes. B) Heatmap illustrating Z scores representing reduced expression levels of genes associated with the cholesterol biosynthesis pathway. Red and blue correspond to up- and down-regulation, respectively. C) Bar graphs display the class average of lysophosphatidylethanolamines (LPE, left) and lysophosphatidylcholines (LPC, right) in Calu-3 cells infected with SNV (blue), ANDV (red), or mock (gray) at 24- and 48-hours post-infection (hpi). D) The graphs show the triglyceride (TG) bonds and TG carbon levels present in the mock and infected Calu-3 cells at 24 and 48 hpi. Quantitative data are presented as mean ± standard deviation. Statistical analysis was performed using ANOVA, followed by Tukey’s post hoc test *, P < 0.05; **, P < 0.01; ***, P < 0.001). (TIF) [file ppat.1013401.s003.tif]

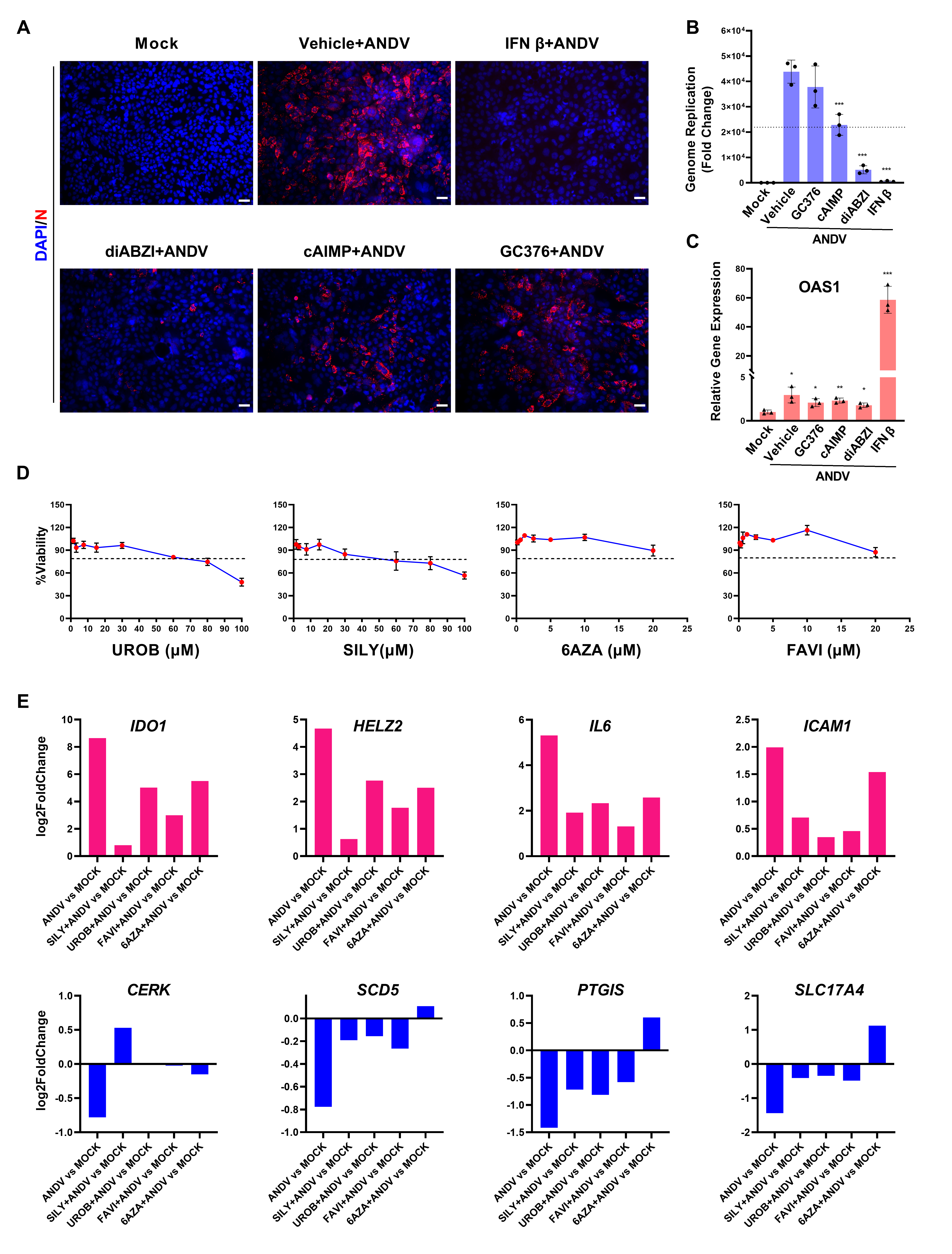

Supplement: S4 Fig — A) Immunofluorescence images of ANDV-infected Calu-3 cells treated with vehicle or various drug compounds at 24 hpi. Red = N protein. Scale bar = 25μm. B) The graph shows the levels of viral genome replication at 24 hpi in response to indicated drug compounds. C) The graph represents relative gene expression levels of the innate immune gene OAS1 in response to treatment with indicated drug compounds. D) The graphs illustrate the dose-response viability assay of indicated drug compounds in Calu-3 cells at 48 hours post-treatment. E) The bar charts show the varying Log2(Fold Change) values of indicated genes in ANDV-infected, as well as drug-treated infected, cells at 48hpi. Quantitative data are presented as mean ± standard deviation. Statistical analysis was performed using ANOVA, followed by Tukey’s post hoc test (*, P < 0.05; **, P < 0.001). ANDV = Andes virus; OAS1 = oligoadenylate synthetase 1; ANOVA = analysis of variance. (TIF) [file ppat.1013401.s004.tif]

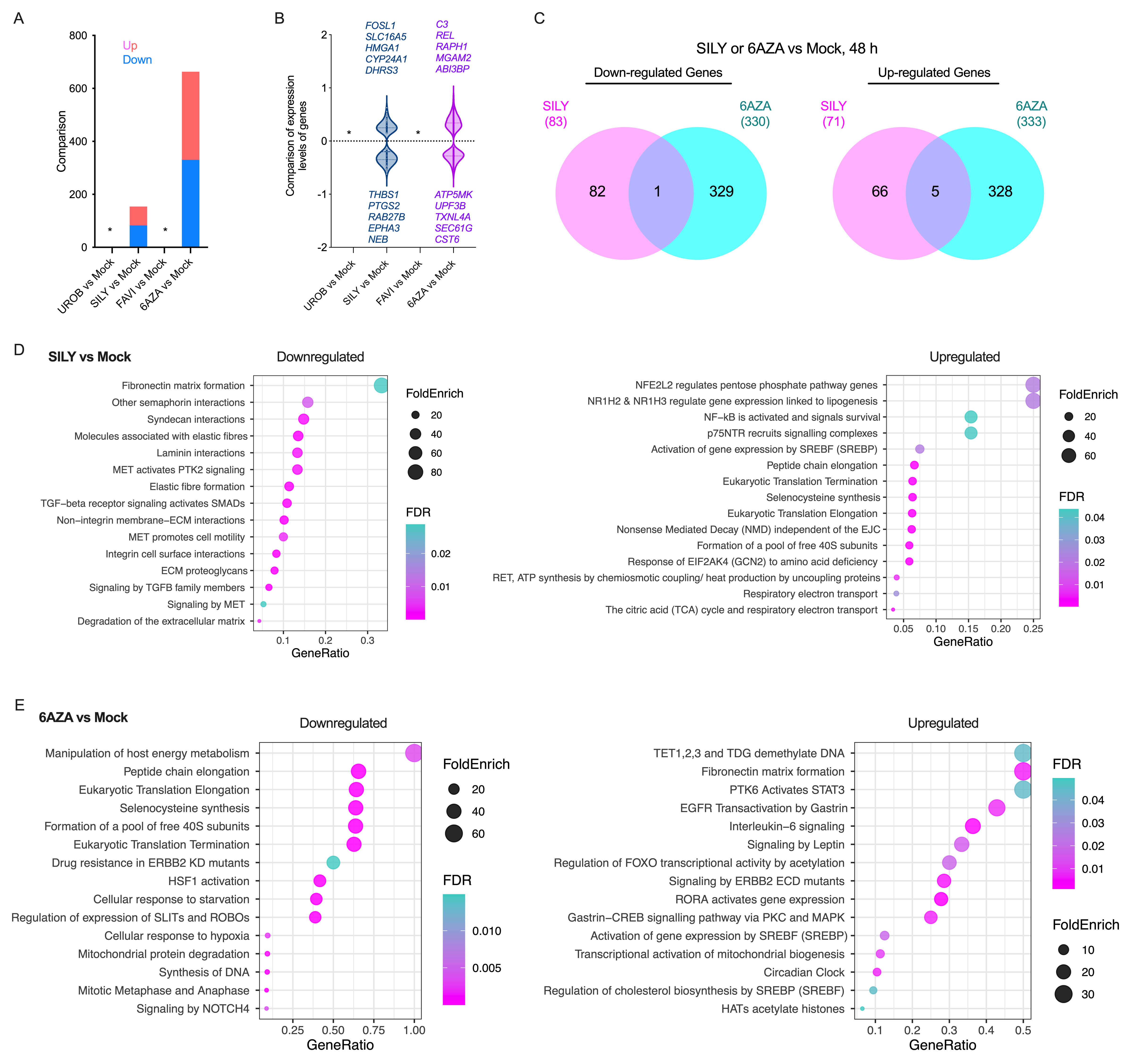

Supplement: S5 Fig — A) Bar chart shows the number of downregulated (blue) and upregulated (red) DEGs in Calu-3 control cells treated with Urolithin B (UROB), Silymarin (SILY), Favipiravir (FAVI), or 6-Azauridine (6AZA) at 48 hours post-drug alone treatment compared to vehicle-treated (Mock) cells. Asterisks indicate conditions with no significant DEGs. B) Violin plot shows patterns of differential gene expression levels in Calu-3 control cells upon drug treatments. The 5 most down- or up-regulated DEGs (padj < 0.01) were displayed. Asterisks indicate conditions with no significant DEGs. C) Venn diagrams illustrate the number of common and distinct genes downregulated (left) and upregulated (right) in Calu3 control cells treated with SILY (pink) or 6AZA (cyan) compared to mock. D, E) Dot plots represent most overrepresented Reactome pathways among downregulated (left) and upregulated (right) genes following treatment with SILY or 6AZA, compared to mock. (TIFF) [file ppat.1013401.s005.tiff]
